# A Tutorial Review of Statistical Snapshot Detectors for GNSS/RAIM Fault Detection: Unified Derivations and Detector Relationships

**DOI:** 10.3390/s26154938

**Published:** 2026-08-04

**Authors:** Penggao Yan, Baoshan Song, Yuan Li, Li-Ta Hsu

**Affiliations:** Department of Aeronautical and Aviation Engineering, The Hong Kong Polytechnic University, Hong Kong, China; baoshan.song@connect.polyu.hk (B.S.); yuaen.li@connect.polyu.hk (Y.L.)

**Keywords:** GNSS, RAIM, fault detection, parity space, solution separation, likelihood-ratio test, projection

## Abstract

The receiver autonomous integrity monitoring (RAIM) and Global Navigation Satellite System (GNSS) fault detection literature uses a group of statistical detectors that are often introduced with different names, coordinate systems, and derivation styles. This makes it difficult for new researchers to determine whether two methods use different information or only express the same inconsistency through different statistics. This paper provides a detector-centered tutorial review of statistical snapshot fault detection with a unified whitened linearized model. The chi-squared detector, parity-space detector, Baarda w-test, range comparison detector, jackknife detector, solution separation detector, and generalized likelihood-ratio test are derived with consistent notation. For each detector, the statistic construction, null and alternative distributions, threshold rule, and minimum detectable bias (MDB) are presented. A relationship map was developed to distinguish exact equivalence, projection relations and linear transformations among these detectors. An illustrative validation was then conducted with a real satellite geometry collected in an urban environment and synthetic Gaussian faults. The results verify the relationship checks, detection-probability behavior, and MDB calculations in a reproducible setting.

## 1. Introduction

Global Navigation Satellite System (GNSS) positioning has become an essential source of positioning, navigation, and timing (PNT) information for aviation, intelligent transportation, autonomous systems, geodesy, and many other PNT applications. However, GNSS positioning can be affected by satellite faults [1,2], receiver-level measurement faults and outliers [3,4,5], multipath and non-line-of-sight propagation [6,7], interference and spoofing [8,9], and environment-dependent modeling errors [10,11]. To make positioning reliable, it is important to detect faulty measurements before a navigation solution is used. This requirement motivated receiver autonomous integrity monitoring (RAIM) and broader integrity-monitoring techniques [12,13,14,15,16].

Detector design remains a central component of integrity-monitoring algorithms. Although modern integrity-monitoring methods may use more complex architectures [10], adopt data-driven approaches [17], or be extended to sensor-fusion applications [18,19], many of them are still constructed or interpreted with reference to classical statistical detectors. For researchers newly entering GNSS integrity monitoring, a persistent difficulty is that these detectors are distributed across different communities, coordinate systems, and naming conventions. For example, the range comparison method introduced an early navigation-domain leave-one-out predicted-range consistency check [20,21]; the jackknife detector [22] organizes the same leave-one-out prediction residual as a modern GNSS/RAIM testing procedure with explicit statistic construction and multiple-testing control. Baarda’s local test comes from geodetic quality control and data snooping [3,4,5], but in the unified residual/parity formulation it is closely connected to the range-comparison statistics [21,23]. The jackknife detector [22] and solution separation [12,24] share a subset-solution structure, but they express the resulting inconsistency in different representations: jackknife uses prediction error in the observation space, whereas solution separation uses separation in the solution/state space [25]. Without a common notation, these links are difficult to identify. Consequently, the literature does not always make clear whether a given method represents a distinct detector, an alternative representation of the same statistic, or a local projection of the same residual information.

Existing studies have already made substantial contributions by reviewing or surveying common integrity-monitoring schemes. For example, RAIM-centered reviews have summarized the development of receiver autonomous integrity monitoring, availability, protection concepts, and future RAIM opportunities [26]. A broader GNSS-integrity line has reviewed the evolution of integrity methods and protection-level concepts beyond a single RAIM formulation [10]. The literature then expands toward application- and system-centered integrity problems, including urban GNSS positioning [6,7], GNSS/INS integrity for autonomous vehicles [16], multi-source fusion navigation [27], localization integrity beyond standalone GNSS [15], and sensor integrity across broader domains [28]. Threat-specific and data-driven reviews further address GNSS jamming and spoofing threats [8,9] and machine-learning-assisted methods [17]. These studies are valuable for positioning RAIM and GNSS integrity within a broader research landscape, but most of them do not treat the detector itself as the central object of study.

A smaller group of papers has examined detector-level relations more directly. Brown’s baseline global positioning system (GPS) RAIM work [21] compared residual, parity, and range-comparison methods, showing that selected classical detectors can be equivalent under matched assumptions. Joerger et al. compared residual-based RAIM and solution separation and developed a parity-space interpretation of solution-separation monitoring [13]. Residual-based RAIM geometry and failure-mode slope have also been analyzed through the measurement and residual subspaces [29]. El-Mowafy compared observation-level and state-estimation-level fault detection formulations in GNSS quality control [23]. Zhao et al. interpreted solution separation through a parity/null-space projection [30]. Liu et al. later connected residual chi-squared separation with solution-separation-type criteria in a fault-exclusion setting [31]. However, these studies only provide partial bridges. A unified guide on how classical detector statistics are derived, how their hypotheses and thresholds are formed, and how their relationships can be understood with one notation is still missing.

To address this gap, this paper reviews the main statistical detectors that have shaped GNSS/RAIM fault detection, including chi-squared [32], parity-space [2], Baarda *w*-test [3], range-comparison [20,21], jackknife detector [22], solution separation [12,24], and the likelihood-ratio test (LRT)/generalized LRT (GLRT). This paper restricts the discussion to the snapshot Gaussian setting with the single fault assumption. The snapshot setting isolates within-epoch measurement redundancy and removes temporal-model differences from the detector-statistic comparison, while the common Gaussian model keeps the stochastic assumptions fixed across detectors. The single fault assumption defines a candidate family over the *n* measurement-coordinate directions: the active index and bias magnitude remain unknown, while the one-active-coordinate structure controls the complexity of the alternative for the unified derivation. The resulting framework serves as a reference layer for interpreting more complex integrity-monitoring methods.

**Figure 1:**
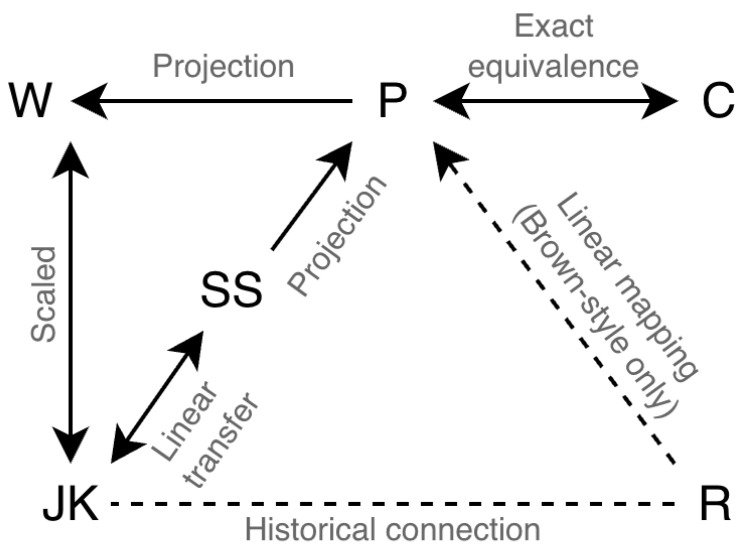
Overview of the relationships among the six detector representations considered in this paper: C, chi-squared detector; P, parity-space detector; W, Baarda *w*-test; R, range comparison detector; JK, jackknife detector; and SS, solution separation detector. The relationships are derived and discussed in Section 4.

We first introduce a unified linearized model and notation then derive the detector statistics, null and alternative distributions, thresholds, and minimum detectable biases (MDB) in the same model. Based on these derivations, we justify the relationship map in Figure 1, distinguishing equivalence, projection, transformation, and framework-level connections among the detectors. We further use a likelihood-ratio perspective to explain why several classical statistics arise from the same underlying hypothesis-testing problem. Simulations were conducted to verify the detection properties of the derived statistics. In this way, this paper provides a unified detector-centered reference that reduces the notational and conceptual burden for new researchers entering GNSS integrity monitoring. The main contributions of this work are summarized as follows.

1. A unified derivation of major statistical detectors in a common whitened model is provided, including the chi-squared detector, parity-space detector, Baarda *w*-test, range comparison detector, jackknife detector, solution separation detector, and GLRT view.
2. A relationship map is established to distinguish exact equivalence, linear transformation, and projection relation among detectors.
3. A simulation study with real GNSS geometry and synthetic noise and bias was conducted, verifying the properties of these detectors in a reproducible way.

The remainder of the paper is organized as follows. Section 2 introduces the unified linearized model and the detector taxonomy. Section 3 derives the detector statistics and their hypothesis-testing quantities. Section 4 summarizes the relationship map among the detector representations. Section 5 explains the same statistics from a likelihood-ratio perspective. Section 6 connects the reviewed statistics to their typical roles and representative use scenarios. Section 7 provides an illustrative validation with real GNSS geometry and synthetic Gaussian faults. Section 8 concludes the paper.

## 2. Unified Snapshot Fault Detection Framework and Detector Taxonomy

To compare the main statistical snapshot detectors within one consistent mathematical setting, this section first fixes a common linear model, a common fault hypothesis, and a common representation vocabulary. It then summarizes how the major detector names used in the literature are organized in this work. This section provides the shared notation and structural map needed by the later sections.

### 2.1. Unified Snapshot Fault Detection Framework

Consider a generalized linear system for GNSS positioning:

(1) y=Gx+ξ,ξ∼N(0,R),

where $y\in R^{n}$ denotes a linearized measurement vector, $G\in R^{n\times m}$ is the design (geometry) matrix in east, north, up coordinates with a clock component for each constellation, $x\in R^{m}$ is the unknown state vector or state correction vector, $\xi \in R^{n}$ is the nominal measurement error, and $R\in R^{n\times n}$ is the corresponding covariance matrix. In this paper, (1) serves as a common statistical entrance rather than as a restriction to one specific observation type. For example, it covers the common pseudorange positioning model, and it can also represent other linearized GNSS positioning problems, such as double-difference pseudorange positioning, after the corresponding observation transformation and covariance propagation are specified. This formulation keeps the model broad enough for several common positioning settings while preserving the same detector-oriented statistical core.

In fault detection problems, redundancy is one of the most important assumptions. We assume that G has full column rank and that the number of measurements exceeds the state dimension,

(2) rank(G)=m,n>m.

The redundancy degree is then

(3) q=n−m.

Only when q>0 does the model contain enough redundant information for snapshot fault detection.

To simplify the derivations, we introduce the whitened version of the general linear model in (1) and use it as the default coordinate system throughout the remainder of this work. Let $R=LL^{⊤}$ be the Cholesky factorization of the symmetric positive-definite matrix R, and define

(4) $$z=L^{-1}y,\;H=L^{-1}G,\;v=L^{-1}\xi .$$

Then (1) becomes

(5) z=Hx+v,v∼N(0,I).

This whitened model removes covariance-weight notation from the later detector derivations and makes the relationships among residual, parity, leave-one-out, and likelihood-based views easier to compare directly.

In this paper, we focus on **snapshot single-measurement fault** detection. For the whitened model, the no-fault and faulty hypotheses are written as

(6) $$H_{0}:z=Hx+v,$$

(7) $$H_{1,k}:z=Hx+be_{k}+v,$$

where $e_{k}\in R^{n}$ denotes the vector whose *k*th entry is one and whose other entries are zero, and *b* is an unknown deterministic bias magnitude.

Two projection matrices will be used repeatedly. The orthogonal projector onto the column space of H is

(8) $$P_{H}=H{\left(H^{⊤}H\right)}^{-1}H^{⊤},$$

and the complementary projector is

(9) $$S=I-P_{H}.$$

The matrix S extracts the measurement inconsistency component that cannot be explained by the state estimate. As a result, it will appear naturally in the chi-squared detector, the parity-space detector, and derivations in Section 3, Section 4 and Section 5.

At this point, it is helpful to distinguish three spaces that will be reused throughout Section 3, Section 4 and Section 5. The first is the *observation space*, where the whitened measurement vector z lives. The second is the *solution/state space*, where the estimated state x and the subset-solution differences used by solution separation live. The third is the *parity/null space*,

(10) $$\mathrm{Null}\left(H^{⊤}\right)=\left\{u\in R^{n}:H^{⊤}u=0\right\},$$

which is the canonical inconsistency subspace in the observation domain.

### 2.2. Detector Taxonomy and Terminology Mapping

This section aims to establish a consistent terminology for the main statistical snapshot detectors so that the subsequent derivations can be presented as a coherent tutorial rather than as isolated summaries. Table 1 aligns common names of detectors in the literature with the preferred names adopted in this work, and Table 2 summarizes the properties of these detectors in the unified framework in Section 2.1.

Notably, two separate descriptors are used in Table 2. *Statistic scope* characterizes the primitive statistic before aggregation: a global statistic summarizes the inconsistency of the full snapshot, a local statistic is indexed by a measurement, fault direction, or solution component. The *detection workflow used in this work* instead identifies the aggregation, threshold policy, and final alarm rule adopted in this paper. A local statistic can therefore remain a specified-direction local test or be aggregated into a family-wise global workflow. The details of the workflow will be illustrated in Section 3 and Section 4. The role of the GLRT/LRT in this work is to interpret the statistical structure of the detectors. Robust, learning-based, sequential, exclusion-oriented, and protection-level-oriented methods remain outside the core scope of this work, even when some adjacent studies use partially related terminology.

## 3. Derivation and Properties of Classical Detectors

**Figure 2:**
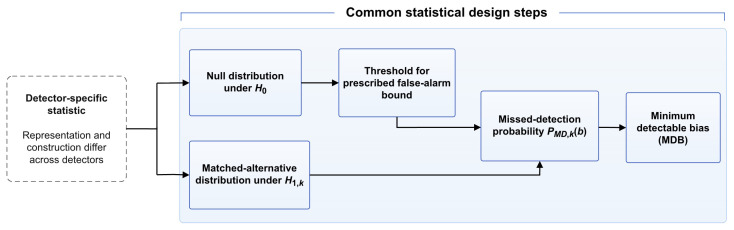
Common statistical design steps used after a detector-specific statistic has been constructed.

Based on the unified model in Section 2.1, this section derives the six core detectors considered in this paper. Although the detectors differ in representation and statistic construction, their statistical design logic follows a common pattern. Once the null and matched-alternative distributions of a detector statistic have been established, the remaining steps are shared: choose a threshold for a prescribed false alarm bound, express the corresponding missed detection probability, and then solve the MDB. Figure 2 summarizes this sequence.

Despite this common design logic, the relevant conditions or specifications, construction and decision steps, and threshold-selection rules must still be stated for each detector. Table 3 therefore summarizes these elements before the detailed derivations. Throughout this section, global and local describe the primitive statistic before aggregation, whereas the detection workflow identifies how those statistics are combined and thresholded to produce the final decision.

This section is organized as follows: the first six subsections derive six detector families: chi-squared, parity-space, Baarda, range comparison, Jackknife, and solution separation. Each establishes its own statistic, relevant hypotheses, distribution, and decision construction. The final Minimum Detectable Bias subsection does not introduce an additional detector; instead, it brings these detector-specific ingredients together to define detection probability and MDB under explicitly stated complete-test contracts.

### 3.1. Chi-Squared Detector

The chi-squared detector [32] is the baseline global consistency test for the whitened linearized model. Let

(11) $$A=H^{⊤}H,$$

(12) $$K=A^{-1}H^{⊤},$$

and the all-in-view least-squares solution is given by

(13) $$\hat{x}=A^{-1}H^{⊤}z=Kz.$$

Using the residual-maker projector $S=I-H{\left(H^{⊤}H\right)}^{-1}H^{⊤}$, the residual vector is

(14) $$r=z-H\hat{x}=\left(I-H{\left(H^{⊤}H\right)}^{-1}H^{⊤}\right)z=Sz.$$

The chi-squared detector statistic is given by

(15) $$T_{R}=r^{⊤}r=z^{⊤}Sz.$$

where the last equality follows from the symmetry and idempotence of S. Because S is the orthogonal projector onto the residual subspace, it removes the component of z that can be fitted by the model Hx and retains only the component unexplained by the column space of H. The chi-squared detector therefore measures the energy of this unexplained component.

For $H_{0}$, we have z=Hx+v with v∼N(0,I). Since SH=0, the residual vector becomes

(16) r=Sz=S(Hx+v)=Sv.

Therefore, r is Gaussian with mean zero and covariance

(17) $$\mathrm{Cov}\left(r\right)=\mathrm{Cov}\left(Sv\right)=S\;\mathrm{Cov}\left(v\right)\;S^{⊤}=SIS^{⊤}=S^{2}=S,$$

where the last equality uses the symmetry and idempotence of S. Because S is an orthogonal projector with rank q=n−m, the residual vector has only *q* independent components in the residual subspace. Consequently, the quadratic form $T_{R}$ follows a chi-squared distribution with *q* degrees of freedom:

(18) $$T_{R}∼\chi_{q}^{2}.$$

For a prescribed false alarm probability α, the threshold is chosen as the upper-tail chi-squared threshold

(19) $$\gamma_{R}=\chi_{q,1-\alpha }^{2},$$

where $\chi_{q,1-\alpha }^{2}$ denotes the (1−α) quantile of the central chi-squared distribution with *q* degrees of freedom. The test rejects $H_{0}$ at significance level α when

(20) $$T_{R}>\gamma_{R}.$$

This chi-squared detector will serve as the global reference for all later detector representations in Section 3.

### 3.2. Parity-Space Detector

The parity-space detector [2] uses the same inconsistency information as the chi-squared detector, but it expresses that information in explicit null-space coordinates. Let $Q\in R^{n\times q}$ be an orthonormal basis of $\mathrm{Null}(H^{⊤})$ such that

(21) $$Q^{⊤}Q=I_{q},\;Q^{⊤}H=0,\;QQ^{⊤}=S.$$

The parity vector is

(22) $$p=Q^{⊤}z,$$

and the parity-space detector statistic is

(23) $$T_{P}=p^{⊤}p.$$

Because Q spans the null space of $H^{⊤}$, the parity vector directly parameterizes the inconsistency subspace introduced in Section 2.1. The parity-space detector therefore does not create a new source of information. Rather, it rewrites the same residual inconsistency in null-space coordinates. Although r is written in *n* observation coordinates, its covariance S has rank q=n−m. The parity vector p expresses the same inconsistency in *q* orthonormal coordinates with identity covariance For $H_{0}$, providing a nonredundant representation that simplifies distributional calculations and makes the projected measurement-bias direction $Q^{⊤}e_{k}$ explicit. Using $QQ^{⊤}=S$, the residual and parity statistics satisfy

(24) $$T_{P}=z^{⊤}QQ^{⊤}z=z^{⊤}Sz=T_{R}.$$

Accordingly, the parity-space detector is not a different detector in the sense of using a new information source. It is the same global consistency test written in canonical null-space coordinates and therefore inherits the null distribution in (18), threshold in (19), and decision rule in (20).

### 3.3. Baarda *w*-Test

The Baarda *w*-test [3] is the one-dimensional local test used in the classical data-snooping setting. In the original geodetic quality-control literature [3], this test is organized as a single-direction statistical test and is commonly interpreted through an *F*-test viewpoint. In the unified notation of this paper, the same detector can be written as a standardized residual test. Reusing the fault-direction notation in (7), $e_{k}$ denotes the *k*th canonical measurement direction, and the local statistic is defined by

(25) $$w_{k}=\frac{e_{k}^{⊤}r}{\sqrt{S_{kk}}}.$$

The normalization term $S_{kk}$ removes the measurement-dependent residual variance and converts the local inconsistency into a comparable scalar test statistic. In this sense, the Baarda *w*-test is the local one-dimensional residual inconsistency after standardization.

For $H_{0}$, as shown in (17), r∼N(0,S). Therefore, $e_{k}^{⊤}r$ is a linear scalar projection of a Gaussian vector and is also Gaussian. Its mean and variance are

(26) $$E\left[e_{k}^{⊤}r\right]=e_{k}^{⊤}E\left[r\right]=0,\;\mathrm{Var}\left(e_{k}^{⊤}r\right)=e_{k}^{⊤}\mathrm{Cov}\left(r\right)e_{k}=e_{k}^{⊤}Se_{k}=S_{kk}.$$

Thus, provided $S_{kk}>0$, we have

(27) $$w_{k}∼N\left(0,1\right).$$

Equivalently,

(28) $$w_{k}^{2}∼\chi_{1}^{2}.$$

In Baarda’s original framing, the latter is also consistent with the one-dimensional *F*-test view with infinite denominator degrees of freedom. In the remainder of this subsection, the detector will be written in this quadratic form.

For a specified measurement direction *k*, let $\alpha_{0}$ denote the local false alarm probability. The corresponding threshold is

(29) $$\gamma_{w}=\chi_{1,1-\alpha_{0}}^{2}.$$

This threshold satisfies $P_{H_{0}}\left(w_{k}^{2}>\gamma_{w}\right)=\alpha_{0}$ for the specified one-dimensional test. The local statistics are correlated across measurement directions because

(30) $$\mathrm{Cov}\left(w_{i},w_{j}\right)=\frac{S_{ij}}{\sqrt{S_{ii}S_{jj}}}.$$

Consequently, when all local statistics are inspected through

(31) $$M_{w}=\max_{k}w_{k}^{2},$$

the probability $P_{H_{0}}\left(M_{w}>\gamma_{w}\right)$ is generally not equal to $\alpha_{0}$. In this paper, $M_{w}$ with the local threshold is used only as an inspection quantity that illustrates this distinction; it is not treated as an independent global workflow in the performance comparison. If a maximum-based Baarda alarm is required, its threshold must instead be selected to control the family-wise false alarm probability, for example through a conservative multiple-testing rule or a correlation-aware numerical calibration.

### 3.4. Range Comparison Detector

Range comparison [20] is one of the earliest leave-one-out prediction-residual ideas in the navigation community. By removing the *k*th measurement, a subset solution is computed from the remaining observations, and the deleted measurement is then predicted from that subset solution. A test statistic is constructed by differencing the *k*th measurement and its prediction. To express this leave-one-out operation, define the deletion matrix

(32) $$L_{k}=I-e_{k}e_{k}^{⊤},$$

whose diagonal entries are all one except the *k*th entry, which is zero. The corresponding subset least-squares solution is

(33) $${\hat{x}}^{\left(k\right)}={\left(H^{⊤}L_{k}H\right)}^{-1}H^{⊤}L_{k}z,$$

and the predicted deleted measurement is

(34) $${\hat{z}}_{k}^{\left(k\right)}=h_{k}{\hat{x}}^{\left(k\right)},$$

where $h_{k}$ denotes the *k*th row of H. The scalar range-comparison residual is then

(35) $$RC_{k}=z_{k}-{\hat{z}}_{k}^{\left(k\right)}=z_{k}-h_{k}{\hat{x}}^{\left(k\right)}.$$

This quantity is the classical left-out prediction error in the observation space. In the taxonomy of Table 2, each $RC_{k}$ is a local statistic. The detection workflow is straightforward: construct $RC_{k}$ for each candidate measurement and inspect which deleted-measurement comparison produces the largest inconsistency. For example, one may define

(36) $$M_{RC}=\max_{k}\left|RC_{k}\right|,$$

and treat the maximizing direction as the primary suspicious-measurement candidate for further scrutiny. In this historical scalar form [20], however, the method is mainly a prediction-comparison idea rather than a fully standardized modern detector package. For that reason, this subsection does not assign an independent thresholded test to the raw scalar construction. The next subsection writes the same navigation-domain leave-one-out prediction residual in jackknife notation and equips it with an explicit statistical testing workflow.

### 3.5. Jackknife Detector

The term jackknife is used for a recent GNSS/RAIM formulation that keeps the same prediction residual as (35), while adding a systematic detector-level treatment: explicit residual construction, null distribution, and multiple-testing control [22]. Reusing the deleted-solution notation in (32), the jackknife residual is defined by

(37) $$J_{k}=z_{k}-h_{k}{\hat{x}}^{\left(k\right)}=RC_{k}.$$

To follow the original jackknife construction more closely, we first rewrite the subset solution in operator form. Define the deleted-solution matrix

(38) $$K^{\left(k\right)}={\left(H^{⊤}L_{k}H\right)}^{-1}H^{⊤}L_{k},$$

so that

(39) $${\hat{x}}^{\left(k\right)}=K^{\left(k\right)}z.$$

The corresponding predicted full measurement vector based on the deleted solution is

(40) $${\tilde{z}}^{\left(k\right)}=H{\hat{x}}^{\left(k\right)}={\tilde{P}}^{\left(k\right)}z,\;{\tilde{P}}^{\left(k\right)}=HK^{\left(k\right)}.$$

Therefore, the deleted-measurement residual vector associated with the *k*th subset is

(41) $$z-{\tilde{z}}^{\left(k\right)}=\left(I-{\tilde{P}}^{\left(k\right)}\right)z.$$

Since

(42) $$\left(I-{\tilde{P}}^{\left(k\right)}\right)H=H-H{\left(H^{⊤}L_{k}H\right)}^{-1}H^{⊤}L_{k}H=0,$$

substituting z=Hx+v into (41) gives

(43) $$z-{\tilde{z}}^{\left(k\right)}=\left(I-{\tilde{P}}^{\left(k\right)}\right)v.$$

Let ${\tilde{p}}_{k}^{\left(k\right)}$ denote the *k*th row of $I-{\tilde{P}}^{\left(k\right)}$. Since the jackknife residual in (37) is the *k*th row of $z-{\tilde{z}}^{\left(k\right)}$, (37) can be written as

(44) $$J_{k}={\tilde{p}}_{k}^{\left(k\right)}v=\sum_{j=1}^{n}{\tilde{p}}_{k,j}^{\left(k\right)}v_{j}.$$

It is shown that the jackknife residual is a linear combination of the whitened measurement errors. In the present Gaussian linearized model, (44) provides the natural starting point for its null distribution.

For $H_{0}$, $J_{k}$ is Gaussian with mean zero and variance

(45) $$\sigma_{J_{k}}^{2}=\mathrm{Var}\left(J_{k}\right)={\tilde{p}}_{k}^{\left(k\right)}{\tilde{p}}_{k}^{(k)T}=\sum_{j=1}^{n}{\left({\tilde{p}}_{k,j}^{\left(k\right)}\right)}^{2}.$$

Therefore,

(46) $$J_{k}∼N\left(0,\sigma_{J_{k}}^{2}\right).$$

The corresponding standardized statistic is

(47) $${\tilde{J}}_{k}=\frac{J_{k}}{\sigma_{J_{k}}}∼N\left(0,1\right),\;{\tilde{J}}_{k}^{2}∼\chi_{1}^{2}.$$

Each ${\tilde{J}}_{k}$ is a local statistic. The detection workflow used in this work aggregates these statistics over all candidate indices through the family-wise maximum test defined below.

We first restate the general no-fault/null-fault pair in (6) and (7) for the specific deleted-measurement test indexed by *k*:

(48) $$\begin{array}{cc} H_{0,k}: & \mathrm{No}\;\mathrm{failure}\;\mathrm{in}\;\mathrm{the}\;k\mathrm{th}\;\mathrm{measurement},\\ H_{1,k}: & A\;\mathrm{failure}\;\mathrm{in}\;\mathrm{the}\;k\mathrm{th}\;\mathrm{measurement}. \end{array}$$

The corresponding origin test rejects $H_{0,k}$ when

(49) $$|J_{k}|>\sigma_{J_{k}}\Phi^{-1}\left(1-\alpha_{0}/2\right),$$

where $\alpha_{0}$ is the false alarm probability of the individual two-sided test. Equivalently, the same local decision rule can be written in standardized quadratic form as

(50) $${\tilde{J}}_{k}^{2}>\gamma_{J,\mathrm{local}},\;\gamma_{J,\mathrm{local}}=\chi_{1,1-\alpha_{0}}^{2}.$$

In practice, this local test is carried out for every candidate deleted measurement, which immediately creates a multiple-testing problem. In such a case, the Type I error can be inflated. Thus, the jackknife literature uses the Bonferroni correction [33] to formalize the workflow-level hypotheses as

(51) $$\begin{array}{cc} H_{0}: & \mathrm{No}\;\mathrm{failure}\;\mathrm{in}\;\mathrm{the}\;n\;\mathrm{measurements},\\ H_{1}: & \mathrm{At}\;\mathrm{least}\;\mathrm{one}\;\mathrm{failure}\;\mathrm{in}\;\mathrm{the}\;n\;\mathrm{measurements}, \end{array}$$

In this test, $H_{0}$ is rejected if any local statistic satisfies

(52) $$|J_{k}|>\sigma_{J_{k}}\Phi^{-1}\left(1-\frac{\tau }{2n}\right),$$

at the significance level $\alpha^{*}$, where τ is the prescribed upper bound on the family-wise false alarm probability $\alpha^{*}$. Equivalently, one may write the corrected rule as

(53) $${\tilde{J}}_{k}^{2}>\gamma_{J},\;\gamma_{J}=\chi_{1,1-\tau /n}^{2}.$$

and then reject $H_{0}$ if this condition holds for any candidate index *k*. In this corrected-test interpretation, the local false alarm probability of each individual test is $\alpha_{0}=\tau /n$.

### 3.6. Solution Separation Detector

Solution separation [12,24] expresses the inconsistency in the solution/state space instead of the observation space. Reusing the definition of the all-in-view least-squares solution in (13) and the solution obtained after excluding the *k*th measurement in (39), the solution separation vector is

(54) $$d_{k}=\hat{x}-{\hat{x}}^{\left(k\right)}=\left(K-K^{\left(k\right)}\right)z.$$

For $H_{0}$, the whitened observation model is z=Hx+v. Since

(55) $$KH=I,\;K^{\left(k\right)}H={\left(H^{⊤}L_{k}H\right)}^{-1}H^{⊤}L_{k}H=I,$$

(54) reduces to

(56) $$d_{k}=\left(K-K^{\left(k\right)}\right)v.$$

Therefore, solution separation is also a linear combination of the measurement errors, but expressed in the solution/state space rather than directly in the observation space.

In the solution separation literature, the hypothesis testing is performed in the east, north, and up components of $d_{k}$ in parallel. Let $\ell_{q}$, with q=1,2,3, denote the projection vector that selects the east, north, and up components, respectively, and define the corresponding directional statistic

(57) $$\Delta_{k,q}=\ell_{q}^{⊤}d_{k}=\ell_{q}^{⊤}\left(K-K^{\left(k\right)}\right)z.$$

For $H_{0}$, (56) gives

(58) $$\Delta_{k,q}=\ell_{q}^{⊤}\left(K-K^{\left(k\right)}\right)v,$$

so the scalar solution-separation statistic is Gaussian with mean zero and variance

(59) $$\sigma_{\Delta_{k,q}}^{2}=\ell_{q}^{⊤}\left(K-K^{\left(k\right)}\right){\left(K-K^{\left(k\right)}\right)}^{⊤}\ell_{q}.$$

Therefore,

(60) $$\Delta_{k,q}∼N\left(0,\sigma_{\Delta_{k,q}}^{2}\right).$$

The normalized scalar statistic is

(61) $${\tilde{\Delta }}_{k,q}=\frac{\Delta_{k,q}}{\sigma_{\Delta_{k,q}}}.$$

For $H_{0}$,

(62) $${\tilde{\Delta }}_{k,q}∼N\left(0,1\right),\;{\tilde{\Delta }}_{k,q}^{2}∼\chi_{1}^{2}.$$

Each directional statistic ${\tilde{\Delta }}_{k,q}$ is local to a candidate measurement and state component. The solution-separation detection workflow is similar to the Jackknife workflow: it uses the same corrected hypotheses in (51). The difference is that, for each candidate measurement, solution separation produces three directional monitors, one for each of the east, north, and up components. In integrity-monitoring practice, the horizontal and vertical components are usually treated differently at the architecture level, so the family-wise false alarm budget is commonly split into a horizontal part and a vertical part rather than distributed uniformly across all three directional statistics [12,13]. The family-wise budget may be redistributed differently depending on the application, provided that the resulting thresholds are updated consistently. With the allocation in the integrity monitoring convention, a Bonferroni-style corrected test rejects $H_{0}$ if any directional statistic satisfies

(63) $$\left|{\tilde{\Delta }}_{k,q}\right|>\Phi^{-1}\left(1-\frac{\tau_{hor}}{2\cdot 2n}\right),\;k=1,\ldots ,n,\;q=1,2,$$

for the east and north components and

(64) $$\left|{\tilde{\Delta }}_{k,3}\right|>\Phi^{-1}\left(1-\frac{\tau_{ver}}{2\cdot n}\right),\;k=1,\ldots ,n,$$

for the up component, where $\tau =\tau_{hor}+\tau_{ver}$ is the prescribed upper bound on the family-wise false alarm probability. Equivalently, one may use the squared-statistic form

(65) $${\tilde{\Delta }}_{k,q}^{2}>\gamma_{SS,q},\;\gamma_{SS,q}=\left\{\begin{array}{cc} \gamma_{SS,hor}=\chi_{1,1-\tau_{hor}/\left(2n\right)}^{2}, & q=1,2,\\ \gamma_{SS,ver}=\chi_{1,1-\tau_{ver}/n}^{2}, & q=3. \end{array}\right.$$

Solution separation is used in this paper solely for detection. Exclusion logic, protection levels, and RAIM risk-allocation issues [12,13] are intentionally left outside the main line of this paper.

### 3.7. Minimum Detectable Bias

The preceding subsections established the statistics, null distributions, thresholds, and aggregation rules that define the alarm constructions. This subsection centralizes their responses to the common single-measurement alternative, the resulting detection and missed-detection probabilities, and the derivation of the MDB. This subsection aims to make explicit that an MDB belongs to a complete thresholded test or workflow alarm, rather than to a statistic formula or detector name in isolation.

#### 3.7.1. Complete-Test Definition and Decision Scope

For a detector or workflow *D* and the direction-specific alternative $H_{1,k}\left(b\right)$ defined in (7), define the complete-test specification

(66) $$C_{D,k}=\left(H_{1,k}\left(b\right),\;T_{D},\;\gamma_{D},\;s_{D},\;P_{D}^{★},\;\Pi_{D}\right),$$

where $T_{D}$ is the statistic, $\gamma_{D}$ is its threshold, $s_{D}$ specifies the rejection sidedness, $P_{D}^{★}$ is the target detection probability, and $\Pi_{D}$ specifies any threshold allocation, candidate set, or aggregation policy. When a single statistic directly produces the alarm, $\Pi_{D}=\Pi_{\mathrm{id}}$ denotes the identity policy with no allocation or cross-statistic aggregation. Let

(67) $$A_{D}\;\left(T_{D}\left(z\right);\gamma_{D},s_{D},\Pi_{D}\right)\in \left\{0,1\right\}$$

denote the resulting alarm. In this paper, the magnitude-based definition below is applied only to sign-invariant tests. For the direction-specific alternative $H_{1,k}\left(b\right)$ and target detection probability $P_{D}^{★}$, define

(68) $$MDB_{D,k}\left(C_{D,k}\right)=\inf\left\{|b|:{\mathrm{Pr}}_{H_{1,k}\left(b\right)}\;\left[A_{D}\;\left(T_{D}\left(z\right);\gamma_{D},s_{D},\Pi_{D}\right)=1\right]\geq P_{D}^{★}\right\}.$$

Thus, an MDB depends jointly on every component in (66), rather than on the statistic formula alone.

This definition admits two distinct decision scopes: (1) A specified-direction local-test MDB, denoted $MDB_{D,k}^{\mathrm{loc}}$, applies one complete scalar test to a fixed direction *k*. (2) A workflow MDB applies the final alarm, which may be produced either by a single global statistic, denoted $MDB_{D,k}^{\mathrm{wf},g}$, or by aggregating multiple candidate-specific local or directional tests, denoted $MDB_{D,k}^{\mathrm{wf},a}$. For $H_{1,k}\left(b\right)$, the index *k* identifies the true biased measurement, whereas the decision scope identifies the alarm whose power is evaluated. Although the workflow alarm itself need not be indexed by *k*, its detection probability and MDB are evaluated For $H_{1,k}\left(b\right)$ and therefore remain direction-conditioned. For each fixed direction *k*, each MDB is a scalar bias magnitude. A direction-free scalar would require an additional best-, worst-, or otherwise aggregated direction policy.

For any complete test, define its detection probability as

(69) $$P_{D,D,k}\left(b\right)={\mathrm{Pr}}_{H_{1,k}\left(b\right)}\;\left[A_{D}\;\left(T_{D}\left(z\right);\gamma_{D},s_{D},\Pi_{D}\right)=1\right].$$

The Type II error probability is therefore

(70) $$P_{\mathrm{MD},D,k}\left(b\right)={\mathrm{Pr}}_{H_{1,k}\left(b\right)}\;\left[A_{D}\;\left(T_{D}\left(z\right);\gamma_{D},s_{D},\Pi_{D}\right)=0\right]=1-P_{D,D,k}\left(b\right).$$

If the test statistic satisfies

(71) $$T_{D}∼\chi_{\nu_{D}}^{2}\;\left(\lambda_{D}\left(b\right)\right),$$

and uses an upper-tail rejection region with threshold $\gamma_{D}$, the required noncentrality $\lambda_{D}^{★}$ is defined by

(72) $$\mathrm{Pr}\;\left[\chi_{\nu_{D}}^{2}\left(\lambda_{D}^{★}\right)>\gamma_{D}\right]=1-F_{\chi_{\nu_{D}}^{2}\left(\lambda_{D}^{★}\right)}\left(\gamma_{D}\right)=P_{D}^{★}.$$

Based on these definitions, the following two subsubsections derive the residual/parity single-global-alarm MDB as an analytic reference and the matched specified-direction local-test MDBs used for direct comparison. The aggregated Jackknife and solution-separation workflows are evaluated separately in Section 7.3 through their stated alarm policies, empirical false-alarm probabilities, and detection-probability curves; their workflow MDBs are not reported.

#### 3.7.2. Residual/Parity Single-Global-Alarm MDB

For the residual/parity single-global-alarm workflow, the complete-test specification is

(73) $$C_{R/P,k}^{\mathrm{wf},g}=\left(H_{1,k}\left(b\right),\;T_{R}\left(=T_{P}\right),\;\gamma_{R},\;\mathrm{upper}\;\mathrm{tail},\;P_{D}^{★},\;\Pi_{\mathrm{id}}\right).$$

The equality $T_{R}=T_{P}$ follows from (24); this statistic is the final decision object. The global threshold $\gamma_{R}=\chi_{q,1-\alpha }^{2}$ is defined in (19), and no threshold allocation or cross-statistic aggregation is applied. Its alarm is

(74) $$A_{R/P}^{\mathrm{wf},g}=1\;\left\{T_{R}>\gamma_{R}\right\}.$$

For the fixed-direction alternative $H_{1,k}\left(b\right)$ in (7), substituting the observation model into the residual representation in (14) gives

(75) $$r=S\left(Hx+be_{k}+v\right)=bSe_{k}+Sv.$$

Accordingly, the detector statistic becomes noncentral chi-squared:

(76) $$T_{R}∼\chi_{q}^{2}\;\left(\lambda_{R,k}\left(b\right)\right),\;\lambda_{R,k}\left(b\right)={∥bSe_{k}∥}^{2}$$

Using the symmetry and idempotence of S,

(77) $$\lambda_{R,k}\left(b\right)=b^{2}{\left(Se_{k}\right)}^{⊤}\left(Se_{k}\right)=b^{2}e_{k}^{⊤}S^{⊤}Se_{k}=b^{2}e_{k}^{⊤}Se_{k}=b^{2}S_{kk}.$$

Here, $S_{kk}$ is the proportion of the *k*th bias direction retained in the residual subspace. With the global threshold $\gamma_{R}$, the missed-detection probability is

(78) $$P_{\mathrm{MD},R/P,k}\left(b\right)=\mathrm{Pr}\left[T_{R}\leq \gamma_{R}|H_{1,k}\left(b\right)\right]=F_{\chi_{q}^{2}\left(\lambda_{R,k}\left(b\right)\right)}\left(\gamma_{R}\right).$$

Define $\lambda_{R}^{★}$ by

(79) $$1-F_{\chi_{q}^{2}\left(\lambda_{R}^{★}\right)}\left(\gamma_{R}\right)=P_{D}^{★}.$$

The corresponding direction-conditioned result satisfies

(80) $$\lambda_{R}^{★}={\left(MDB_{R/P,k}^{\mathrm{wf},g}\right)}^{2}S_{kk},$$

and therefore

(81) $$MDB_{R/P,k}^{\mathrm{wf},g}=\sqrt{\frac{\lambda_{R}^{★}}{S_{kk}}}.$$

Residual and parity share this value because (24) gives the same final statistic and decision rule. If $S_{kk}=0$, the direction is absorbed by the fitted model and this value is infinite. Because this quantity belongs to a *q*-dimensional single-global alarm, it is not part of the matched local-test comparison below and is not numerically ranked against the local-test MDBs as a measure of intrinsic detector sensitivity in Section 7.

#### 3.7.3. Matched Specified-Direction Local-Test MDB

The direct local comparison uses a different, matched contract. For Baarda and Jackknife, let $U_{D,k}$ denote the signed standardized scalar for fixed direction *k*, with $U_{w,k}=w_{k}$ from (25) and $U_{J,k}={\tilde{J}}_{k}$ from (47). For solution separation, the component *q* is fixed in advance and $U_{SS,k,q}={\tilde{\Delta }}_{k,q}$ from (61). The decision statistic is the square of the corresponding signed scalar, while two-sidedness is defined with respect to that signed scalar. The common complete-test specification is

(82) $$C_{D,k}^{\mathrm{loc}}=\left(H_{1,k}\left(b\right),\;U_{D,k}^{2},\;\gamma_{\mathrm{loc}},\;\mathrm{two}\text{-}\mathrm{sided},\;P_{D}^{★},\;\Pi_{\mathrm{id}}\right),$$

For Baarda and Jackknife, this contract is indexed by *k*. For solution separation, its exact specialization is $C_{SS,k,q}^{\mathrm{loc}}$ with decision statistic $U_{SS,k,q}^{2}$; *q* is fixed before observing the data, and $\Pi_{\mathrm{id}}$ excludes selection, threshold allocation, or aggregation across either *k* or *q*. Equivalently, the two-sided alarm is written in squared form as

(83) $$A_{D,k}^{\mathrm{loc}}=1\;\left\{U_{D,k}^{2}>\gamma_{\mathrm{loc}}\right\}.$$

For solution separation, (83) specializes to $A_{SS,k,q}^{\mathrm{loc}}=1\left\{U_{SS,k,q}^{2}>\gamma_{\mathrm{loc}}\right\}$. The common local false alarm probability $\alpha_{0}$ gives

(84) $$\gamma_{\mathrm{loc}}=\chi_{1,1-\alpha_{0}}^{2}.$$

A two-sided rejection in the signed scalar *U* is therefore equivalent to an upper-tail rejection in $U^{2}$. For the same target $P_{D}^{★}$, define the common required noncentrality $\lambda_{\mathrm{loc}}^{★}$ by

(85) $$1-F_{\chi_{1}^{2}\left(\lambda_{\mathrm{loc}}^{★}\right)}\left(\gamma_{\mathrm{loc}}\right)=P_{D}^{★}.$$

This matched threshold and target, rather than the Jackknife Bonferroni threshold or the solution-separation H/V allocation, define the direct local-test comparison. Because the three local statistics are already standardized, their alternative responses can be expressed directly through one scalar recipe. Whenever a signed scalar statistic satisfies

(86) U∼N(cb,1),

where *c* is its sensitivity to the specified bias, its squared form satisfies

(87) $$U^{2}∼\chi_{1}^{2}\left(\lambda \right),\;\lambda =c^{2}b^{2}.$$

Equation (85) fixes the noncentrality required by the common threshold and target, whereas (87) gives the noncentrality induced by a bias of magnitude |b|. For this upper-tail noncentral chi-squared test, the detection probability increases with λ. Hence, the target is reached when $c^{2}b^{2}\geq \lambda_{\mathrm{loc}}^{★}$, and the minimum detectable magnitude is attained at the boundary

(88) $$\lambda_{\mathrm{loc}}^{★}=c^{2}{\left(MDB^{\mathrm{loc}}\right)}^{2}.$$

For c≠0, $MDB^{\mathrm{loc}}$ is nonnegative, so taking the positive square root of (88) gives $|c|MDB^{\mathrm{loc}}=\sqrt{\lambda_{\mathrm{loc}}^{★}}$. Solving for the bias magnitude therefore yields

(89) $$MDB^{\mathrm{loc}}=\frac{\sqrt{\lambda_{\mathrm{loc}}^{★}}}{\left|c\right|}.$$

Baarda *w*-test:

For $H_{1,k}\left(b\right)$ in (7), the observation model is $z=Hx+be_{k}+v$. Substituting this model into the residual representation in (14) and then into the Baarda statistic in (25) gives

(90) $$\begin{array}{cc} w_{k}\; & =\frac{e_{k}^{⊤}r}{\sqrt{S_{kk}}}=\frac{e_{k}^{⊤}Sz}{\sqrt{S_{kk}}}\\ \; & =\frac{e_{k}^{⊤}SHx+be_{k}^{⊤}Se_{k}+e_{k}^{⊤}Sv}{\sqrt{S_{kk}}}\\ \; & =b\sqrt{S_{kk}}+\frac{e_{k}^{⊤}Sv}{\sqrt{S_{kk}}}, \end{array}$$

where the last equality uses SH=0 and $e_{k}^{⊤}Se_{k}=S_{kk}$. The noise term $\frac{e_{k}^{⊤}Sv}{\sqrt{S_{kk}}}$ is Gaussian with zero mean. Using the symmetry and idempotence of S, its variance is

(91) $$\begin{array}{cc} \mathrm{Var}\;\left(\frac{e_{k}^{⊤}Sv}{\sqrt{S_{kk}}}\right)\; & =\frac{e_{k}^{⊤}S\mathrm{Cov}\left(v\right)S^{⊤}e_{k}}{S_{kk}}\\ \; & =\frac{e_{k}^{⊤}SS^{⊤}e_{k}}{S_{kk}}=\frac{e_{k}^{⊤}Se_{k}}{S_{kk}}=1. \end{array}$$

Therefore,

(92) $$w_{k}∼N\;\left(b\sqrt{S_{kk}},1\right).$$

Applying the shared recipe, we have

(93) $$c=\sqrt{S_{kk}},\;MDB_{w,k}^{\mathrm{loc}}=\sqrt{\frac{\lambda_{\mathrm{loc}}^{★}}{S_{kk}}}.$$

Jackknife local test:

The deleted solution does not use the *k*th observation. From the definition of $L_{k}$ in (32), we have

(94) $$L_{k}e_{k}=\left(I-e_{k}e_{k}^{⊤}\right)e_{k}=e_{k}-e_{k}\left(e_{k}^{⊤}e_{k}\right)=0.$$

Using the deleted-solution matrix $K^{\left(k\right)}$ defined in (38), it follows that

(95) $$K^{\left(k\right)}e_{k}={\left(H^{⊤}L_{k}H\right)}^{-1}H^{⊤}L_{k}e_{k}=0.$$

Consequently, substituting $H_{1,k}\left(b\right)$ from (7) into the deleted-solution operator in (39) and using the exact-fit identity $K^{\left(k\right)}H=I$ in (55) gives

(96) $$\begin{array}{cc} {\hat{x}}^{\left(k\right)} & =K^{\left(k\right)}z\\ \; & =K^{\left(k\right)}\left(Hx+be_{k}+v\right)\\ \; & =K^{\left(k\right)}Hx+bK^{\left(k\right)}e_{k}+K^{\left(k\right)}v\\ \; & =x+K^{\left(k\right)}v. \end{array}$$

Substituting (96) into the jackknife residual defined in (37), and using $z_{k}=e_{k}^{⊤}z$ and $e_{k}^{⊤}H=h_{k}$, gives

(97) $$\begin{array}{cc} J_{k} & =z_{k}-h_{k}{\hat{x}}^{\left(k\right)}\\ \; & =e_{k}^{⊤}\left(Hx+be_{k}+v\right)-h_{k}\left(x+K^{\left(k\right)}v\right)\\ \; & =h_{k}x+b+e_{k}^{⊤}v-h_{k}x-h_{k}K^{\left(k\right)}v\\ \; & =b+\left(e_{k}^{⊤}-h_{k}K^{\left(k\right)}\right)v\\ \; & =b+{\tilde{p}}_{k}^{\left(k\right)}v. \end{array}$$

Here, ${\tilde{p}}_{k}^{\left(k\right)}$ is the *k*th deleted-residual coefficient vector used in (44); the variance of the noise term ${\tilde{p}}_{k}^{\left(k\right)}v$ is $\sigma_{J_{k}}^{2}$ from (45). Hence,

(98) $$J_{k}∼N\;\left(b,\sigma_{J_{k}}^{2}\right),\;{\tilde{J}}_{k}∼N\;\left(\frac{b}{\sigma_{J_{k}}},1\right).$$

Applying the shared recipe, we have

(99) $$c=\frac{1}{\sigma_{J_{k}}},\;MDB_{J,k}^{\mathrm{loc}}=\sigma_{J_{k}}\sqrt{\lambda_{\mathrm{loc}}^{★}}.$$

Solution-separation local test:

For $H_{1,k}\left(b\right)$ in (7), substituting the observation model into the solution-separation vector defined in (54) gives

(100) $$\begin{array}{cc} d_{k} & =\left(K-K^{\left(k\right)}\right)z\\ \; & =\left(K-K^{\left(k\right)}\right)\left(Hx+be_{k}+v\right)\\ \; & =\left(KH-K^{\left(k\right)}H\right)x+b\left(K-K^{\left(k\right)}\right)e_{k}\\ \; & \;+\left(K-K^{\left(k\right)}\right)v\\ \; & =b\left(K-K^{\left(k\right)}\right)e_{k}+\left(K-K^{\left(k\right)}\right)v, \end{array}$$

where the last equality uses the exact-fit identities $KH=K^{\left(k\right)}H=I$ in (55). Recall from (57) that $\ell_{q}$ selects a fixed east, north, or up component. Projecting (100) onto this component gives

(101) $$\begin{array}{cc} \Delta_{k,q} & =\ell_{q}^{⊤}d_{k}\\ \; & =b\ell_{q}^{⊤}\left(K-K^{\left(k\right)}\right)e_{k}+\ell_{q}^{⊤}\left(K-K^{\left(k\right)}\right)v\\ \; & =b\eta_{k,q}+\ell_{q}^{⊤}\left(K-K^{\left(k\right)}\right)v, \end{array}$$

where

(102) $$\eta_{k,q}=\ell_{q}^{⊤}\left(K-K^{\left(k\right)}\right)e_{k}$$

is the mean response of the selected component to a unit bias in direction *k*. The noise term in (101) is zero-mean Gaussian with the variance $\sigma_{\Delta_{k,q}}^{2}$ defined in (59). Therefore,

(103) $$\Delta_{k,q}∼N\;\left(b\eta_{k,q},\sigma_{\Delta_{k,q}}^{2}\right),\;{\tilde{\Delta }}_{k,q}∼N\;\left(\frac{b\eta_{k,q}}{\sigma_{\Delta_{k,q}}},1\right).$$

Applying the shared recipe, we have

(104) $$c=\frac{\eta_{k,q}}{\sigma_{\Delta_{k,q}}},\;MDB_{SS,k,q}^{\mathrm{loc}}=\frac{\sigma_{\Delta_{k,q}}}{|\eta_{k,q}|}\sqrt{\lambda_{\mathrm{loc}}^{★}}.$$

## 4. Relationship Map

Section 3 derived the detector statistics one by one, and the derivations already revealed several connections among them. For example, the chi-squared detector and the parity-space detector lead to the same quadratic statistic in the whitened model. The Baarda *w*-test is constructed from a standardized component of the residual vector. The range comparison detector and the jackknife detector use the same scalar leave-one-out residual in (37). The solution separation detector then compares the all-in-view solution with the deleted-measurement solution in the solution/state space. This section justifies the relationship map previewed in Figure 1 by making these detector-to-detector relationships explicit in the common whitened model.

To justify this map, Section 4.1 first derives the relationship between the chi-squared detector and the parity-space detector and then establishes the covariance-normalized Brown-style [21] block range-comparison relationship to the same global statistic. Section 4.2 then derives the relationships among the local Baarda, range-comparison, jackknife, and Solution Separation statistics and the parity-space representation. Notably, the map connects detector statistics and their representations; it does not encode the final detection workflows. Workflow-level decision equivalence additionally requires the same aggregation rule, threshold, and decision object.

### 4.1. Global-Statistic Relationships

#### 4.1.1. Chi-Squared Detector and Parity-Space Detector

The exact equivalence between the chi-squared detector and the parity-space detector has already been given in (23) and (24). We restate this relationship here:

(105) $$T_{P}=p^{⊤}p=z^{⊤}QQ^{⊤}z=z^{⊤}Sz=T_{R}.$$

where p is the parity vector, Q is an orthonormal basis of $\mathrm{Null}(H^{⊤})$, and $QQ^{⊤}=S$. All these definitions can be found in Section 3. The relationship revealed in (105) is an exact statistic equivalence in the common whitened model, which follows the classical residual/parity construction used in the RAIM literature [2,21].

#### 4.1.2. Brown-Style Block Range Comparison and Parity-Space Detector

The range comparison detector can also be connected to the global residual/parity relationship but only when a full block of range-comparison residuals is normalized by its covariance [21]. Let q=n−m. After reordering the observations if needed, partition

(106) $$z=\left[\begin{array}{c} z_{1}\\ z_{2} \end{array}\right],\;H=\left[\begin{array}{c} H_{1}\\ H_{2} \end{array}\right],$$

where $z_{1}\in R^{m\times 1}$, $z_{2}\in R^{q\times 1}$, $H_{1}\in R^{m\times m}$ is nonsingular, and $H_{2}\in R^{q\times m}$. The base-subset solution is

(107) $${\hat{x}}_{1}=H_{1}^{-1}z_{1},$$

and the corresponding block range-comparison residual is

(108) $$f_{B}=z_{2}-H_{2}{\hat{x}}_{1}=\left[\begin{array}{cc} -H_{2}H_{1}^{-1} & I_{q} \end{array}\right]z=B_{RC}z.$$

The identity $B_{RC}H=0$ shows that the rows of $B_{RC}$ belong to $\mathrm{Null}(H^{⊤})$. Moreover, the right block $I_{q}$ makes $B_{RC}$ the full row rank. For $H_{0}$, the block residual therefore satisfies

(109) $$f_{B}=B_{RC}v∼N\left(0,R_{f}\right),\;R_{f}=B_{RC}B_{RC}^{⊤}=I_{q}+H_{2}H_{1}^{-1}H_{1}^{-T}H_{2}^{⊤}.$$

Because $B_{RC}$ is a full row rank, $R_{f}$ is positive definite and admits the symmetric inverse square root $R_{f}^{-1/2}$. The covariance-normalized block statistic is consequently

(110) $$T_{RC}^{B}=f_{B}^{⊤}R_{f}^{-1}f_{B}∼\chi_{q}^{2}\;\mathrm{for}\;H_{0}.$$

To establish the exact relationship to the parity statistic, define the covariance-whitened row-basis matrix and residual vector as

(111) $${\tilde{B}}_{RC}=R_{f}^{-1/2}B_{RC},\;{\tilde{f}}_{B}=R_{f}^{-1/2}f_{B}={\tilde{B}}_{RC}z.$$

It follows that

(112) $${\tilde{B}}_{RC}H=0,\;{\tilde{B}}_{RC}{\tilde{B}}_{RC}^{⊤}=I_{q}.$$

Thus, the rows of ${\tilde{B}}_{RC}$ form an orthonormal basis of the *q*-dimensional parity/null space. They therefore generate the same orthogonal projector as $Q^{⊤}$:

(113) $${\tilde{B}}_{RC}^{⊤}{\tilde{B}}_{RC}=S.$$

Consequently, the normalized range-comparison statistic is pointwise identical to the residual and parity statistics:

(114) $$\begin{array}{cc} T_{RC}^{B} & ={\tilde{f}}_{B}^{⊤}{\tilde{f}}_{B}=z^{⊤}{\tilde{B}}_{RC}^{⊤}{\tilde{B}}_{RC}z\\ \; & =z^{⊤}Sz=T_{R}=T_{P}. \end{array}$$

Equivalently, because $Q^{⊤}$ and ${\tilde{B}}_{RC}$ are orthonormal row bases of the same subspace, an orthogonal matrix $U\in R^{q\times q}$ exists such that $p=U{\tilde{f}}_{B}$. The raw vectors p and $f_{B}$ therefore contain the same inconsistency information through an invertible linear transform, but their unweighted Euclidean norms are not generally equal. Exact statistic equivalence follows from the covariance-normalized equality in (114), for the common whitened model and the full-rank base-subset condition. If the same aggregation rule and threshold are applied to these identical statistics, their alarm decisions are also identical. This result does not extend to the scalar or maximum range-comparison construction [20] in Section 3.4 without additional conditions.

### 4.2. Local-Statistic Relationships

#### 4.2.1. Baarda *w*-Test and Parity-Space Detector

The Baarda *w*-test statistic has already been defined in (25). To rewrite it in parity coordinates, we reuse $QQ^{⊤}=S$ from (21) and $p=Q^{⊤}z$ from (22). These identities give

(115) $$r=Sz=QQ^{⊤}z=Qp,\;S_{kk}=e_{k}^{⊤}Se_{k}=e_{k}^{⊤}QQ^{⊤}e_{k}={∥Q^{⊤}e_{k}∥}^{2}.$$

Substituting (115) into (25), the Baarda *w*-test statistic can be written as

(116) $$w_{k}=\frac{e_{k}^{⊤}r}{\sqrt{S_{kk}}}=\frac{e_{k}^{⊤}Qp}{\sqrt{e_{k}^{⊤}QQ^{⊤}e_{k}}}=\frac{{\left(Q^{⊤}e_{k}\right)}^{⊤}p}{∥Q^{⊤}e_{k}∥}.$$

Thus the Baarda *w*-test statistic is a normalized projection of the parity vector p onto the direction $Q^{⊤}e_{k}$, which is the component of the *k*th measurement-bias direction that remains in the parity/null space. This explains the P→W edge in Figure 1: the full parity vector determines $w_{k}$, but a single $w_{k}$ cannot recover the full parity vector unless the parity/null space is one-dimensional or the analysis is restricted to that single direction.

#### 4.2.2. Range Comparison Detector and Jackknife Detector

The next relationship is the historical-to-modern connection between the range comparison detector and the jackknife detector. Equation (37) has already established the identity with the same leave-one-out construction:

(117) $$J_{k}=z_{k}-h_{k}{\hat{x}}^{\left(k\right)}=RC_{k}.$$

However, it does not mean that Lee’s range-comparison method [20] already contained the complete modern jackknife testing procedure [22]. Rather, Lee’s range-comparison method provides the prediction-error idea, while the jackknife formulation provides the more systematic modern testing notation, including standardization and family-wise testing.

#### 4.2.3. Jackknife Detector and Solution Separation Detector

Since both the jackknife and solution separation detectors use subset solutions to construct the test statistics, this subsection establishes their algebraic relationship in the common whitened model. From (13), the all-in-view normal equation is

(118) $$A\hat{x}=H^{⊤}z,$$

$A=H^{⊤}H$ has been defined in (11). From (33), the normal equation of the subset solution is

(119) $$H^{⊤}L_{k}H{\hat{x}}^{\left(k\right)}=H^{⊤}L_{k}z.$$

Using $L_{k}=I-e_{k}e_{k}^{⊤}$ from (32), the left-hand matrix becomes

(120) $$H^{⊤}L_{k}H=H^{⊤}\left(I-e_{k}e_{k}^{⊤}\right)H=A-h_{k}^{⊤}h_{k},$$

and the right-hand vector becomes

(121) $$H^{⊤}L_{k}z=H^{⊤}\left(I-e_{k}e_{k}^{⊤}\right)z=H^{⊤}z-h_{k}^{⊤}z_{k}.$$

Substituting (120) and (121) into (119) gives

(122) $$\left(A-h_{k}^{⊤}h_{k}\right){\hat{x}}^{\left(k\right)}=H^{⊤}z-h_{k}^{⊤}z_{k}.$$

Subtracting (122) from (118) gives

(123) $$\begin{array}{cc} A\left(\hat{x}-{\hat{x}}^{\left(k\right)}\right) & =h_{k}^{⊤}z_{k}-h_{k}^{⊤}h_{k}{\hat{x}}^{\left(k\right)}\\ \; & =h_{k}^{⊤}\left(z_{k}-h_{k}{\hat{x}}^{\left(k\right)}\right)\\ \; & =h_{k}^{⊤}J_{k}. \end{array}$$

Therefore,

(124) $$d_{k}=\hat{x}-{\hat{x}}^{\left(k\right)}=A^{-1}h_{k}^{⊤}J_{k}.$$

Intuitively, this relationship reveals that the difference in solutions, induced by excluding the *k*th measurement, is proportional to the perturbation of the *k*th measurement and oriented along the direction $A^{-1}h_{k}^{⊤}$ in the solution space. Indeed, $A^{-1}h_{k}^{⊤}$ is the derivative of the solution $\hat{x}$ with respect to the *k*-th measurement

(125) $$\frac{\;d\hat{x}}{\;dz_{k}}=A^{-1}h_{k}^{⊤}\;,$$

which quantifies how each element in $\hat{x}$ changes in response to a small perturbation in $z_{k}$.

For a given projection vector $\ell_{q}$, the scalar solution separation statistic is

(126) $$\Delta_{k,q}=\ell_{q}^{⊤}d_{k}=\left(\ell_{q}^{⊤}A^{-1}h_{k}^{⊤}\right)J_{k}.$$

If the coefficient $\ell_{q}^{⊤}A^{-1}h_{k}^{⊤}$ is nonzero and the sign of $\Delta_{k,q}$ is retained, $J_{k}$ can be recovered from that selected component.

#### 4.2.4. Baarda *w*-Test and Jackknife Detector

With (124) established, the relationship between the Baarda *w*-test and the jackknife detector becomes clear. Using $A=H^{⊤}H$ from (11) and the residual-maker matrix in (14), the *k*th diagonal entry of S is

(127) $$S_{kk}=e_{k}^{⊤}Se_{k}=e_{k}^{⊤}\left(I-HA^{-1}H^{⊤}\right)e_{k}=1-h_{k}A^{-1}h_{k}^{⊤}.$$

The *k*th residual is given by

(128) $$\begin{array}{cc} r_{k} & =z_{k}-h_{k}\hat{x}\\ \; & =z_{k}-h_{k}{\hat{x}}^{\left(k\right)}-h_{k}\left(\hat{x}-{\hat{x}}^{\left(k\right)}\right)\\ \; & =J_{k}-h_{k}\left(\hat{x}-{\hat{x}}^{\left(k\right)}\right) \end{array}$$

Substituting the solution-separation identity in (124) into (128) gives

(129) $$\begin{array}{cc} r_{k} & =J_{k}-h_{k}A^{-1}h_{k}^{⊤}J_{k}\\ \; & =\left(1-h_{k}A^{-1}h_{k}^{⊤}\right)J_{k}\\ \; & =S_{kk}J_{k}. \end{array}$$

Consequently,

(130) $$w_{k}=\frac{r_{k}}{\sqrt{S_{kk}}}=\sqrt{S_{kk}}J_{k},$$

provided $S_{kk}>0$. Since S is an orthogonal projector, $S_{kk}\geq 0$. For a regular GNSS snapshot geometry with redundant measurements, $S_{kk}$ is usually positive. Therefore, for a fixed measurement index *k* with $S_{kk}>0$, the Baarda *w*-test and the jackknife residual describe the same one-dimensional observation-space inconsistency but with different geometry-dependent scalings. The degenerate case $S_{kk}=0$ would mean that the *k*th measurement-bias direction is entirely absorbed by the fitted state model, or equivalently that the corresponding observation has unit leverage. This is not expected in ordinary redundant GNSS geometries, but it marks the mathematical boundary of the local testability condition.

Taking variances in (129) and using $\mathrm{Var}\left(r_{k}\right)=S_{kk}$ gives

(131) $$\sigma_{J_{k}}^{2}=\frac{1}{S_{kk}},\;{\tilde{J}}_{k}=\frac{J_{k}}{\sigma_{J_{k}}}=\sqrt{S_{kk}}J_{k}=w_{k}.$$

Thus, for the one-deleted-measurement construction, the Baarda statistic and standardized Jackknife statistic are exactly the same scalar, rather than merely proportional statistics.

Nevertheless, this is a statistic-level identity and does not by itself determine the decision policy. The Baarda procedure in this paper applies the local threshold to a specified $w_{k}^{2}$, whereas the jackknife workflow uses a family-wise threshold over all candidate indices. If both representations were instead combined with the same maximum rule and threshold, they would produce identical alarm decisions.

#### 4.2.5. Solution Separation Detector and Parity-Space Detector

The solution separation detector can also be related to the parity-space detector. From (129), the jackknife residual can be written as

(132) $$J_{k}=\frac{r_{k}}{S_{kk}},\;S_{kk}>0.$$

Using r=Qp in (115), the *k*th residual component can be written as

(133) $$r_{k}=e_{k}^{⊤}r=e_{k}^{⊤}Qp={\left(Q^{⊤}e_{k}\right)}^{⊤}p.$$

Following the parity-space fault-mode interpretation used in solution-separation and chi-squared RAIM comparisons [13,34], define the unit direction associated with the *k*th measurement-bias direction in the parity/null space as

(134) $$u_{k}=\frac{Q^{⊤}e_{k}}{∥Q^{⊤}e_{k}∥}=\frac{Q^{⊤}e_{k}}{\sqrt{S_{kk}}},\;S_{kk}>0.$$

Here the second equality follows from $S_{kk}=e_{k}^{⊤}Se_{k}=e_{k}^{⊤}QQ^{⊤}e_{k}={∥Q^{⊤}e_{k}∥}^{2}$ in (115). Therefore, (133) gives

(135) $$u_{k}^{⊤}p=\frac{{\left(Q^{⊤}e_{k}\right)}^{⊤}p}{\sqrt{S_{kk}}}=\frac{r_{k}}{\sqrt{S_{kk}}}=w_{k}=\sqrt{S_{kk}}J_{k}.$$

Thus $u_{k}^{⊤}p$ is not an additional detector statistic. It is the normalized local inconsistency associated with the *k*th measurement-bias direction, written as a one-dimensional projection of the parity vector.

Substituting $J_{k}=\left(u_{k}^{⊤}p\right)/\sqrt{S_{kk}}$ from (135) into (126) gives

(136) $$\Delta_{k,q}=\frac{\ell_{q}^{⊤}A^{-1}h_{k}^{⊤}}{\sqrt{S_{kk}}}u_{k}^{⊤}p.$$

Appendix A shows that $\frac{\left|\ell_{q}^{⊤}A^{-1}h_{k}^{⊤}\right|}{\sqrt{S_{kk}}}$ equals $\sigma_{\Delta_{k,q}}$, the standard deviation of the scalar solution-separation statistic in (59). Therefore, the normalized scalar solution-separation statistic is

(137) $${\tilde{\Delta }}_{k,q}=\frac{\Delta_{k,q}}{\sigma_{\Delta_{k,q}}}=\mathrm{sign}\;\left(\ell_{q}^{⊤}A^{-1}h_{k}^{⊤}\right)u_{k}^{⊤}p=\mathrm{sign}\;\left(\eta_{k,q}\right)w_{k}=\mathrm{sign}\;\left(\eta_{k,q}\right){\tilde{J}}_{k}.$$

For every nondegenerate component with $S_{kk}>0$ and $\eta_{k,q}\neq 0$, it follows pointwise that

(138) $$w_{k}^{2}={\tilde{J}}_{k}^{2}={\tilde{\Delta }}_{k,q}^{2}.$$

When these statistics are paired with the same $H_{1,k}\left(b\right)$, two-sided local threshold $\gamma_{\mathrm{loc}}$, and target $P_{D}^{★}$ defined in Section 3.7, they therefore have identical local power functions and identical specified-direction local-test MDBs. This relationship also indicates that a scalar solution separation statistic can be written as a signed one-dimensional projection of the parity vector along the fault-mode direction $u_{k}$. It is therefore related to the parity-space detector through the same parity/null-space information, but it is not generally the same statistic as the global norm $T_{P}$. This distinction is consistent with the residual-based RAIM versus solution-separation comparisons in [13,34], where solution separation is tied to fault-hypothesis-specific detector mappings rather than to a single global chi-squared norm.

## 5. Likelihood-Ratio Interpretation

Section 4 organized algebraic relationships among the detector statistics. In this section, we use the GLRT view to explain why several statistic forms derived in Section 3 and Section 4 naturally arise with different alternative specifications. In this sense, the GLRT view is a framework interpretation rather than an additional detector in Figure 1.

### 5.1. Parity Reduction and One-Dimensional GLRT

The starting point is again the whitened linearized model in (5),

(139) z=Hx+v,v∼N(0,I).

Applying the parity/null-space basis Q defined in (21) can remove the nuisance state x in (139). This is because $Q^{⊤}H=0$. The parity vector p defined in (22) is therefore given by

(140) $$p=Q^{⊤}z=Q^{⊤}\left(Hx+v\right)=Q^{⊤}v.$$

Since $Q^{⊤}v∼N\left(0,I_{n-m}\right)$, the null hypothesis becomes

(141) $$H_{0}:\;p∼N\left(0,I_{n-m}\right).$$

Consider the single-measurement bias case as in (7),

(142) $$z=Hx+be_{k}+v.$$

In parity/null-space coordinates,

(143) $$\begin{array}{cc} p & =Q^{⊤}\left(Hx+be_{k}+v\right)\\ \; & =bQ^{⊤}e_{k}+Q^{⊤}v. \end{array}$$

Because $Q^{⊤}v∼N\left(0,I_{n-m}\right)$, (143) gives

(144) $$H_{1,k}:\;p∼N\left(bQ^{⊤}e_{k},I_{n-m}\right)=N\left(b\sqrt{S_{kk}}u_{k},I_{n-m}\right),$$

where the second equality reuses the unit parity fault-mode direction $u_{k}$ defined in (134). Therefore, the GLRT can be read as a Gaussian mean-shift test in the parity/null space [35,36].

When $S_{kk}>0$, the Gaussian likelihood for $H_{0}$ is proportional to

(145) $$L_{0}\left(p\right)\propto \exp\left(-\frac{1}{2}p^{⊤}p\right),$$

whereas the likelihood for the single-direction alternative is

(146) $$L_{1,k}\left(b;p\right)\propto \exp\left[-\frac{1}{2}{∥p-b\sqrt{S_{kk}}u_{k}∥}^{2}\right].$$

In this paper, the generalized likelihood ratio is written as the null likelihood divided by the maximized alternative likelihood:

(147) $$\Lambda_{k}\left(p\right)=\frac{\max L_{0}\left(p\right)}{\max_{b}L_{1,k}\left(b;p\right)}=\frac{L_{0}\left(p\right)}{\max_{b}L_{1,k}\left(b;p\right)}.$$

Therefore, a small value of $\Lambda_{k}\left(p\right)$ indicates that the optimized alternative explains the observed parity vector better than the null model. To evaluate the denominator in (147), only $L_{1,k}\left(b;p\right)$ must be maximized with respect to *b*. This maximization is equivalent to minimizing the squared distance

(148) $$D_{k}\left(b\right)={∥p-b\sqrt{S_{kk}}u_{k}∥}^{2}.$$

Equivalently, maximizing the alternative likelihood in the denominator is the same as maximizing $-\frac{1}{2}D_{k}\left(b\right)$. Differentiating this objective gives

(149) $$\frac{\partial }{\partial b}\left[-\frac{1}{2}D_{k}\left(b\right)\right]=\sqrt{S_{kk}}u_{k}^{⊤}p-bS_{kk}.$$

Setting (149) to zero yields

(150) $$\hat{b}=\frac{u_{k}^{⊤}p}{\sqrt{S_{kk}}}.$$

Substituting $\hat{b}$ into (147) gives the following expression:

(151) $$\begin{array}{cc} -2\log\Lambda_{k}\left(p\right) & =p^{⊤}p-{∥p-\hat{b}\sqrt{S_{kk}}u_{k}∥}^{2}\\ \; & =p^{⊤}p-{∥p-\left(u_{k}^{⊤}p\right)u_{k}∥}^{2}\\ \; & =p^{⊤}p-\left[p^{⊤}p-2{\left(u_{k}^{⊤}p\right)}^{2}+{\left(u_{k}^{⊤}p\right)}^{2}u_{k}^{⊤}u_{k}\right]\\ \; & =p^{⊤}p-\left[p^{⊤}p-{\left(u_{k}^{⊤}p\right)}^{2}\right]\\ \; & ={\left(u_{k}^{⊤}p\right)}^{2}, \end{array}$$

where the last reduction uses the unit-vector property $u_{k}^{⊤}u_{k}=1$ from (134). Therefore, the one-dimensional GLRT statistic can be written as

(152) $$T_{\mathrm{GLRT},k}={\left(u_{k}^{⊤}p\right)}^{2}.$$

Using (135), this statistic becomes

(153) $$T_{\mathrm{GLRT},k}={\left(u_{k}^{⊤}p\right)}^{2}=w_{k}^{2},$$

and for $H_{0}$, $T_{\mathrm{GLRT},k}∼\chi_{1}^{2}$. This shows that the Baarda *w*-test statistic is a direct example of the one-dimensional GLRT form under Gaussian noise conditions [36].

### 5.2. GLRT Statistic in Full Parity-Space

The same likelihood-ratio calculation can be stated for a broader bias-vector alternative in the observation space. Instead of specifying a single measurement direction, write

(154) $$z=Hx+\delta +v,\;\delta \in R^{n},$$

where δ denotes an unknown bias vector and is different from the scalar bias *b* used in the single-measurement alternative. Applying the parity/null-space basis gives

(155) $$p=Q^{⊤}z=Q^{⊤}\delta +Q^{⊤}v,$$

and the bias-vector alternative is

(156) $$H_{1,\mathrm{full}}:\;p∼N\left(Q^{⊤}\delta ,I_{n-m}\right),\;Q^{⊤}\delta \in R^{n-m}.$$

The likelihood for $H_{0}$ is still proportional to $\exp(-p^{⊤}p/2)$, whereas the likelihood with this alternative is

(157) $$L_{1,\mathrm{full}}\left(\delta ;p\right)\propto \exp\left[-\frac{1}{2}{∥p-Q^{⊤}\delta ∥}^{2}\right].$$

With the same convention as in (147), the generalized likelihood ratio is

(158) $$\Lambda_{\mathrm{full}}\left(p\right)=\frac{L_{0}\left(p\right)}{\max_{\delta }L_{1,\mathrm{full}}\left(\delta ;p\right)}.$$

The denominator is maximized when the parity-space mean shift matches the observed parity vector, namely, when $Q^{⊤}\hat{\delta }=p$; for example, $\hat{\delta }=Qp$ satisfies this identity because $Q^{⊤}Q=I_{n-m}$. Substituting this maximizing value into the likelihood ratio gives

(159) $$\begin{array}{cc} -2\log\Lambda_{\mathrm{full}}\left(p\right) & =p^{⊤}p-{∥p-Q^{⊤}\hat{\delta }∥}^{2}\\ \; & =p^{⊤}p. \end{array}$$

Thus the GLRT statistic in the parity space is

(160) $$T_{\mathrm{GLRT},\mathrm{full}}=p^{⊤}p=T_{P}.$$

For $H_{0}$, $T_{\mathrm{GLRT},\mathrm{full}}∼\chi_{n-m}^{2}$, which is the same statistic form used by the global parity/null-space test.

From Section 5.1 and Section 5.2, we have seen that the GLRT view is alternative-dependent. A GLRT statistic is not unique until the alternative family has been specified. A known one-dimensional direction gives a squared scalar projection, whereas a parity-space mean shift gives the full quadratic norm. Consequently, a likelihood-ratio formula may explain the origin of a statistic form, but it does not automatically reproduce the full detector workflow, such as normalization, maximum operations, family-wise threshold allocation, or selected solution components.

## 6. Typical Roles and Representative Use Scenarios

The preceding derivations and relationship analysis establish how the reviewed detector statistics are constructed and related. For tutorial readers, Table 4 complements this mathematical view by answering two practical questions: what each statistic checks and in which representative workflow that check appears.

As can be seen, the chi-squared and parity-space detectors begin with the all-in-view observation set: both assess global consistency, using the residual representation and parity/null-space coordinates, respectively. Their representative scenarios place this shared question in classical residual-based RAIM and in parity-coordinate RAIM with explicit fault-mode projection. The Baarda *w*-test narrows the check to the local consistency of the *k*th observation, supporting observation screening and data snooping. Range comparison then tests a held-out pseudorange against the prediction from the remaining observations, and the Jackknife detector organizes the same leave-one-out logic across candidate measurements or fault modes; the corresponding scenarios trace this check from historical range-comparison RAIM to standardized Jackknife RAIM testing workflows. Solution separation shifts the reported quantity to the change in a selected state component under a fault hypothesis, as used in multiple-hypothesis solution-separation (MHSS) RAIM.

## 7. Illustrative Validation with Real GNSS Geometry and Synthetic Faults

Section 3, Section 4 and Section 5 derived the main detector statistics in a common whitened linearized model and related them through projection, leave-one-out, and likelihood-ratio arguments. Section 6 then connected these statistics to representative workflows without treating them as a performance ranking. This section validates the preceding derivations in two parts. The first examines the MDBs of matched specified-direction local tests built from the Baarda, standardized Jackknife, and normalized scalar solution-separation statistics. The second evaluates the final fault-detection workflows under their stated workflow-level false-alarm policies. The purpose is not to benchmark detector performance in real urban faults. Instead, a simulation study with a real GNSS snapshot geometry, synthetic Gaussian noise, and synthetic single-measurement biases was deployed in a reproducible setting. Accordingly, this controlled setting did not evaluate detector behavior on independently labeled real NLOS or multipath measurements.

### 7.1. Experiment Setting

The geometry used in this experiment comes from epoch 3549 of GPS-only data collected in a Hong Kong urban environment and was used only as a realistic snapshot geometry for detector validation. The satellite and receiver positions were converted into the local east, north, and up geometry matrix with a receiver-clock component, following the coordinate convention used in Section 2.1. The finalized geometry matrix used in the simulation is provided in Appendix B. As summarized in Table 5, the number of measurements was n=11, the number of state components was m=4, the redundancy was q=7, and the geometry (design) matrix was full column rank.

An identity covariance was used for the synthetic noise in this setting; therefore, the whitened geometry matrix was the same as the original geometry matrix, and the validation was performed directly in the whitened model defined in (5). The absolute receiver position was used only to construct the geometry matrix; the system state was a delta state around the linearization point. Therefore, x=0 was used in the simulated observations. Let $j=1,\ldots ,N_{\mathrm{MC}}$ denote the Monte Carlo trial index. Samples were generated as

(161) zj=Hx+bek+vj=bek+vj,vj∼N(0,I),

where the bias *b* ranges from 0 to 10 with spacing 0.5. For each bias level, we conducted $N_{\mathrm{MC}}$ = 10,000 Monte Carlo trials.

### 7.2. MDB of Matched Local Tests

This section examines the MDBs of matched specified-direction local tests. In this experiment, the same $H_{1,k}\left(b\right)$, two-sided squared rule, local false alarm probability $\alpha_{0}=0.05$, and target detection probability $P_{D}^{★}=0.95$ were used. These settings give the common threshold $\gamma_{\mathrm{loc}}=\chi_{1,0.95}^{2}$. For each measurement direction *k*, the experiment evaluated $w_{k}^{2}$, ${\tilde{J}}_{k}^{2}$, and every nondegenerate ${\tilde{\Delta }}_{k,q}^{2}$ under the common local-test contract.

Table 6 reports the three detector-specific local-MDB calculations in separate columns, together with the maximum pointwise squared-statistic identity error over the $H_{0}$ Monte Carlo samples. The maximum difference among the local-MDB calculations is $9.50\times 10^{-14}$ whitened standard deviations, and the largest squared-statistic identity error is $6.56\times 10^{-12}$. Thus, the three MDB columns expose the detector-specific calculations, while their numerical agreement confirms the exact relationship in (138); they are not three independent empirical estimates. These values are specified-direction local-test MDBs and are not workflow MDBs.

**Figure 3:**
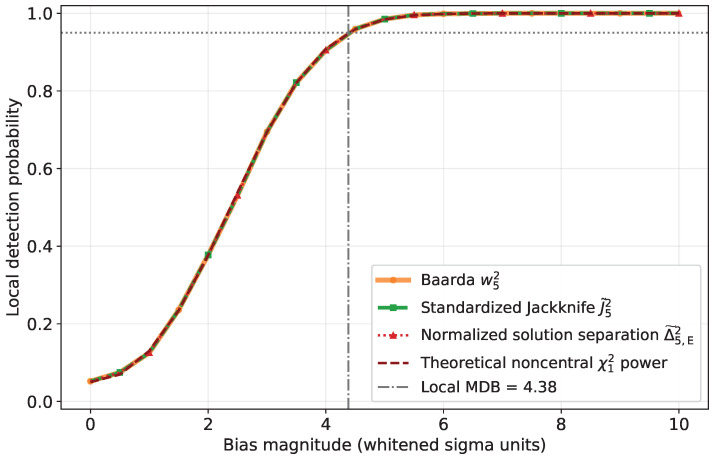
Empirical power curves for the three matched specified-direction local tests for satellite G09 at k=5, plotted separately for $w_{5}^{2}$, ${\tilde{J}}_{5}^{2}$, and ${\tilde{\Delta }}_{5,E}^{2}$, together with their common theoretical power. The curves coincide under the common local threshold. The vertical line indicates their common local-test MDB of 4.38 whitened standard deviations, and the horizontal dotted line represents the target detection probability $P_{D}^{★}=0.95$.

Figure 3 plots the empirical power (or detection probability) separately for $w_{5}^{2}$, ${\tilde{J}}_{5}^{2}$, and the fixed east-component statistic ${\tilde{\Delta }}_{5,E}^{2}$, together with their common theoretical noncentral-$\chi_{1}^{2}$ power. The east component is used only to display one specified solution-separation local test; the other nondegenerate components give the same curve. Before the Monte Carlo simulation, the experiment selected the measurement with median $S_{kk}$ among the 11 directions, namely, satellite G09 at k=5 with $S_{kk}=0.677$. This avoids selecting the easiest or most difficult direction. The three empirical curves coincide pointwise, the maximum absolute empirical–theoretical difference is 0.00768, and their common local MDB is 4.38 whitened standard deviations.

### 7.3. Comparison of Fault-Detection Workflows

This section evaluates the final fault-detection workflows under their stated workflow-level false-alarm policies. Specifically, the chi-squared/parity workflow uses α=0.05, whereas the Jackknife workflow applies the family-wise budget τ=0.05 to the union over all local Jackknife tests. For the solution-separation horizontal/vertical (H/V) workflow, the illustrative allocation uses $\tau_{hor}=0.025$ and $\tau_{ver}=0.025$ with the rule in (65). To separate this integrity-monitoring convention from a detection-only use of solution separation, we also include solution separation max-sensitivity: for each candidate measurement *k*, this variant selects the east, north, or up component with the largest sensitivity $|\ell_{q}^{⊤}A^{-1}h_{k}^{⊤}|$ and applies the family-wise threshold $\chi_{1,1-\tau /n}^{2}$ to the resulting one-dimensional detector family. This variant is not meant to replace the conventional directional detector; it is included only to show what solution separation becomes when the task is restricted to detection only. Table 7 lists the final alarm rule and threshold-allocation policy for each evaluated procedure. For the same reason given in Section 7.2, satellite G09 at k=5 was selected for bias injection.

**Figure 4:**
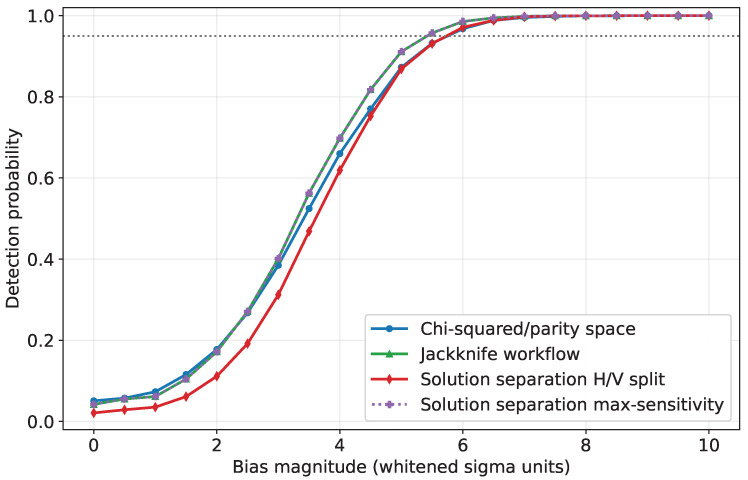
Workflow detection probability versus bias magnitude on the common bias grid. The solution-separation max-sensitivity curve is drawn over the exactly coincident Jackknife workflow curve. The other curves are conditioned on their stated threshold-allocation and aggregation policies and are not an intrinsic sensitivity ranking. The horizontal dotted line represents the target detection probability $P_{D}^{★}=0.95$.

Figure 4 shows the empirical workflow detection probabilities on the common b=0–10 bias grid. The chi-squared/parity curve is close to the Jackknife workflow curve, although the two procedures use different statistic dimensions and thresholds. The Jackknife workflow coincides with the solution-separation max-sensitivity curve because both evaluate the same one-dimensional leave-one-out inconsistency family with the same family-wise rule. The solution-separation H/V curve is lower because the same underlying scalar information is tested through several directional monitors while the family-wise budget is divided between the horizontal and vertical groups. Note that these curves are conditioned on the stated allocation and aggregation policies; they do not establish an intrinsic sensitivity ranking.

**Figure 5:**
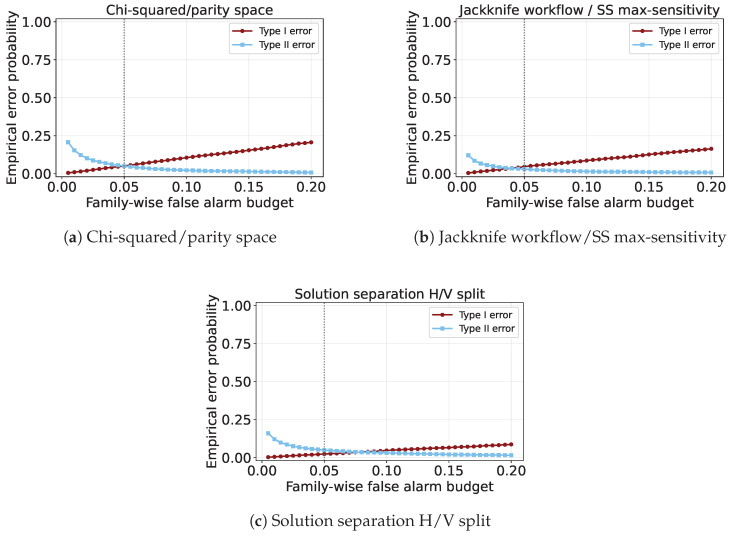
Type I/Type II error against the family-wise false alarm budget for the global workflows.

Figure 5 shows how Type I and Type II errors change with the family-wise false-alarm budget. For all three panels, the same reference bias $b^{\mathrm{ref}}=5.68$ whitened standard deviations was injected into satellite G09 at k=5. This value was obtained by inverting the chi-squared/parity global noncentral-$\chi^{2}$ response at α=0.05 and $P_{D}^{★}=0.95$. For each false-alarm budget, the corresponding complete workflow alarm rule was applied to the resulting samples. The empirical Type II error is the proportion of these samples for which the workflow does not raise an alarm. The common fault magnitude makes the panels directly comparable as policy-conditioned workflow responses, although their different threshold allocations still preclude an intrinsic sensitivity ranking. Within each panel, increasing the false-alarm budget raises the Type I error and lowers the Type II error.

Table 8 reports the empirical false-alarm and missed-detection probabilities at the nominal workflow budget of 0.05. The false-alarm probability is evaluated for $H_{0}$, whereas the missed-detection probability is evaluated for the common G09 reference fault used in Figure 5. These values give the realized error pair for each stated workflow policy. Because the aggregation rules and the resulting empirical false-alarm probabilities are not identical, the table is not used to infer an intrinsic workflow ranking.

Additional sensitivity results for good, normal, and poor satellite geometries in GPS-only and GPS + BeiDou configurations are reported in Appendix C. These additional results retain the same synthetic-noise and single-measurement-bias assumptions used above.

## 8. Conclusions

This paper presented a tutorial review of the main statistical snapshot detectors used in RAIM and GNSS fault detection, including the chi-squared detector, parity-space detector, Baarda *w*-test, range comparison detector, jackknife detector, solution separation detector, and generalized likelihood-ratio test, based on a unified linearized model and notation. The review shows that several detector families are connected through the same residual inconsistency information. The residual and parity-space detectors give the same global quadratic statistic in different coordinates. The range comparison and jackknife detectors express leave-one-out prediction inconsistency, with the jackknife detector providing a more systematic GNSS/RAIM testing workflow. The Baarda *w*-test uses local standardized residual projections, whereas solution separation maps the leave-one-out inconsistency into the solution/state space. The likelihood-ratio discussion further clarifies that different statistic forms arise from different alternative families. A practical-use guide connects these mathematical distinctions to representative RAIM, ARAIM, and geodetic quality-control workflows. Using a real GPS snapshot geometry and synthetic Gaussian faults, the numerical validation confirms the common power and MDB of the matched specified-direction local tests for every measurement direction. It separately reports the final alarm policies, empirical false-alarm probabilities, and common-grid detection-probability curves of the fault-detection workflows. The main value of this tutorial review is to help new researchers trace a detector name back to its statistic, hypothesis, threshold policy, and information representation, rather than mistaking a change of notation or coordinate system for a new source of detection information.

Nevertheless, the scope of this paper is deliberately limited. It does not involve fault exclusion, protection-level computation, robust estimation, learning-based detection, sequential monitoring, or non-Gaussian integrity risk allocation. These topics are important, but they require additional modeling assumptions and decision layers beyond the statistical snapshot detection problem. Future work may examine how the detector-centered relationships studied here extend to exclusion, protection-level, robust-estimation, or non-Gaussian settings.

## Figures and Tables

**Table 1:** Name mapping.

Common Literature Term	Preferred Term in This Work	Statistic Representation/View
Residual-based detector, least-squares residual test, chi-squared detector, global test	Chi-squared detector	Residual representation written in observation-space coordinates
Parity method, parity-space detector, parity vector test	Parity-space detector	Parity/null-space representation
Local test, standardized residual, normalized residual, Baarda *w*-test, data snooping	Baarda *w*-test	Local standardized projection of the observation-space residual representation.
Range comparison method, range comparison detector, leave-one-out range check	Range comparison detector	Leave-one-out prediction-error representation in observation-space coordinates
Jackknife test, jackknife residual, leave-one-out prediction residual	Jackknife detector	Leave-one-out prediction-error representation in observation-space coordinates
Solution separation, subset solution separation	Solution separation detector	Solution/state-space separation representation
LRT, GLRT, likelihood-ratio detector	GLRT/LRT view	Likelihood-ratio representation

**Table 2:** Detector taxonomy.

Detector/Framework	Statistic Scope	Core Statistic or Object	Detection Workflow Used in This Work
Chi-squared detector	Global	$r=(I-P_{H})z$, $T_{R}={∥r∥}^{2}$	Single global test
Parity-space detector	Global	$p=Q^{⊤}z$, $T_{P}={∥p∥}^{2}$	Single global test
Baarda *w*-test	Local	$w_{k}$ or $w_{k}^{2}$	Specified-direction local test
Range comparison detector	Local	$RC_{k}=z_{k}-h_{k}{\hat{x}}^{\left(k\right)}$	Inspection only; no standalone test
Jackknife detector	Local	$J_{k}=z_{k}-h_{k}{\hat{x}}^{\left(k\right)}$	Family-wise maximum test (global)
Solution separation detector	Local	$d_{k}=\hat{x}-{\hat{x}}^{\left(k\right)}$	Family-wise H/V-split test (global)
GLRT/LRT view	Alternative-dependent	Likelihood-ratio object determined by the specified alternative	No fixed detection workflow

**Table 3:** Detector-specific conditions or specifications, construction and decision steps, and threshold selection.

Detector/ Framework	Detector-Specific Condition/Specification	Main Construction and Decision Steps	Threshold Selection in This Work
Chi-squared detector	None beyond Section 2.1.	Fit the all-in-view least-squares solution; form r=Sz and $T_{R}={∥r∥}^{2}$; apply a single global alarm.	Given global false-alarm probability α, use the analytic upper-tail quantile $\gamma_{R}=\chi_{q,1-\alpha }^{2}$.
Parity-space detector	None beyond Section 2.1.	Construct an orthonormal Q; form $p=Q^{⊤}z$ and $T_{P}={∥p∥}^{2}$; apply the same single global alarm.	Since $T_{P}=T_{R}$, inherit the same $\chi_{q}^{2}$ null law, α, and $\gamma_{R}$.
Baarda *w*-test	Specify measurement direction *k* and require $S_{kk}>0$.	Form the residual r; project onto direction *k*; standardize to $w_{k}$; square to $w_{k}^{2}$; make the specified-direction local decision.	Given local false-alarm probability $\alpha_{0}$, use the analytic quantile $\gamma_{w}=\chi_{1,1-\alpha_{0}}^{2}$.
Range comparison detector	For each inspected *k*, require $H^{⊤}L_{k}H$ to be nonsingular, equivalently $S_{kk}>0$.	Delete measurement *k*; compute ${\hat{x}}^{\left(k\right)}$; predict $z_{k}$; form $RC_{k}$; inspect the candidate inconsistency.	No standalone calibrated threshold is assigned to the raw scalar $RC_{k}$ in this work.
Jackknife detector	Every deletion in the candidate family must yield a nonsingular $H^{⊤}L_{k}H$.	Fix the eligible candidate family; compute $J_{k}$ and standardize it to ${\tilde{J}}_{k}$ for every candidate; apply a family-wise alarm.	For the current all-*n* eligible workflow, prescribe family-wise budget τ, allocate τ/n by Bonferroni, and set $\gamma_{J}=\chi_{1,1-\tau /n}^{2}$.
Solution separation detector	For each monitored pair (k,q), require a valid deletion and $\ell_{q}^{⊤}A^{-1}h_{k}^{⊤}\neq 0$.	Fix eligible deletions and monitored horizontal/vertical components; form $d_{k}$; project to $\Delta_{k,q}$ and standardize it to ${\tilde{\Delta }}_{k,q}$; apply a family-wise alarm.	Given $\tau_{hor}$ and $\tau_{ver}$, set $\gamma_{SS,hor}=\chi_{1,1-\tau_{hor}/\left(2n\right)}^{2}$ and $\gamma_{SS,ver}=\chi_{1,1-\tau_{ver}/n}^{2}$; the allocation is an application-level input.
GLRT/LRT view	Specify the alternative signature or family and the nuisance-state treatment.	Specify $H_{0}/H_{1}$; remove or profile the nuisance state; maximize the likelihood; form the alternative-dependent statistic.	No universal threshold or independent GLRT/LRT workflow is specified in this work.

**Table 4:** What the reviewed detector statistics answer and where they are typically used.

Detector Statistic/View	What Question Does It Answer?	Representative Use Scenario
Chi-squared detector	Is the all-in-view observation set globally consistent in the residual representation?	Classical residual-based RAIM all-in-view consistency checks [1].
Parity-space detector	Is the same global inconsistency significant in parity/null-space coordinates?	Parity-coordinate RAIM and explicit fault-mode projection [2,30].
Baarda *w*-test	Is the *k*th observation locally inconsistent?	Observation screening and data snooping in geodetic networks and GNSS quality control [3,23].
Range comparison detector	Does a held-out pseudorange agree with the prediction from the remaining observations?	Historical leave-one-out range-comparison RAIM [20,21].
Jackknife detector	How can leave-one-out consistency checks be standardized across candidate measurements or fault modes?	Jackknife detector and Jackknife RAIM testing workflows [22,25].
Solution separation detector	Does a selected state component change significantly under a fault hypothesis?	MHSS-based RAIM [13,30].

**Table 5:** Summary of the satellite geometry used in the experiment.

Constellation	*n*	*m*	*q*	Rank(H)	$\mathrm{Cond}(H^{⊤}H)$	$S_{\mathrm{kk}}$ Range
GPS	11	4	7	4	16.90	0.528–0.750

**Table 6:** Matched specified-direction local-test MDB and exact-identity evidence for every measurement direction.

*k*	$S_{\mathrm{kk}}$	${\mathrm{MDB}}_{w,k}^{\mathrm{loc}}$	${\mathrm{MDB}}_{J,k}^{\mathrm{loc}}$	${\mathrm{MDB}}_{\mathrm{SS},k,q}^{\mathrm{loc}}$	Maximum Identity Error
1	0.594	4.68	4.68	4.68	$6.39\times 10^{-14}$
2	0.554	4.84	4.84	4.84	$7.11\times 10^{-14}$
3	0.701	4.31	4.31	4.31	$6.56\times 10^{-12}$
4	0.750	4.16	4.16	4.16	$3.31\times 10^{-13}$
5	0.677	4.38	4.38	4.38	$1.56\times 10^{-13}$
6	0.528	4.96	4.96	4.96	$3.64\times 10^{-14}$
7	0.551	4.85	4.85	4.85	$8.35\times 10^{-14}$
8	0.679	4.38	4.38	4.38	$7.90\times 10^{-14}$
9	0.696	4.32	4.32	4.32	$1.83\times 10^{-13}$
10	0.534	4.93	4.93	4.93	$2.22\times 10^{-14}$
11	0.737	4.20	4.20	4.20	$1.95\times 10^{-13}$

**Table 7:** Final alarm rules and threshold policies for the evaluated workflows.

Workflow/Evidence Object	Final Alarm	Threshold Policy
Chi-squared/parity single global test	$T_{R}\left(=T_{P}\right)>\gamma_{R}$	α=0.05
Jackknife workflow	${⋃}_{k}\left\{{\tilde{J}}_{k}^{2}>\gamma_{J}\right\}$	τ=0.05
Solution separation H/V workflow	${⋃}_{k,q}\left\{{\tilde{\Delta }}_{k,q}^{2}>\gamma_{SS,q}\right\}$	$\tau_{hor}=0.025$, $\tau_{ver}=0.025$
Solution separation max-sensitivity workflow	$\max_{k}{\tilde{\Delta }}_{k,q(k)}^{2}>\gamma_{J}$	τ=0.05

**Table 8:** Empirical false-alarm and missed-detection probabilities at the nominal workflow budget of 0.05 for the epoch-3549 G09 reference-fault experiment.

Workflow/Evidence Object	Nominal Workflow Budget	Empirical ${\hat{P}}_{\mathrm{FA}}$	Empirical ${\hat{P}}_{\mathrm{MD}}$ at $b^{\mathrm{ref}}=5.68$
Chi-squared/parity single global test	α=0.05	0.0506	0.0500
Jackknife workflow/solution separation max-sensitivity	τ=0.05	0.0462	0.0305
Solution separation H/V workflow	$\tau_{\mathrm{hor}}=0.025$, $\tau_{\mathrm{ver}}=0.025$	0.0234	0.0493

**Figure A1:**
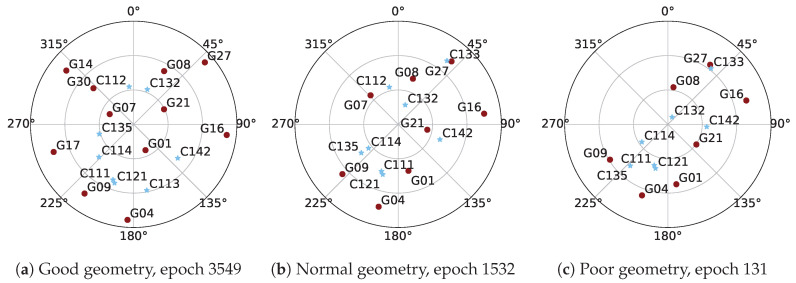
GPS+BeiDou sky plots for the selected good, normal, and poor geometry cases. GPS satellites are shown as circles and BeiDou satellites as star markers. The center is 90° elevation; the inner and outer rings are 60° and 30°, respectively; and the rim is 0°.

**Figure A2:**
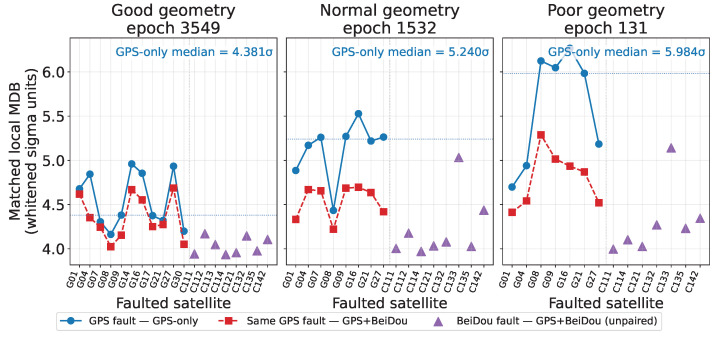
Matched specified-direction local-test MDBs for all eligible fault directions. The blue series gives the GPS-only values, the red dashed series gives the same GPS fault directions after adding BeiDou, and the purple markers give the additional BeiDou fault directions, which have no GPS-only counterpart.

**Figure A3:**
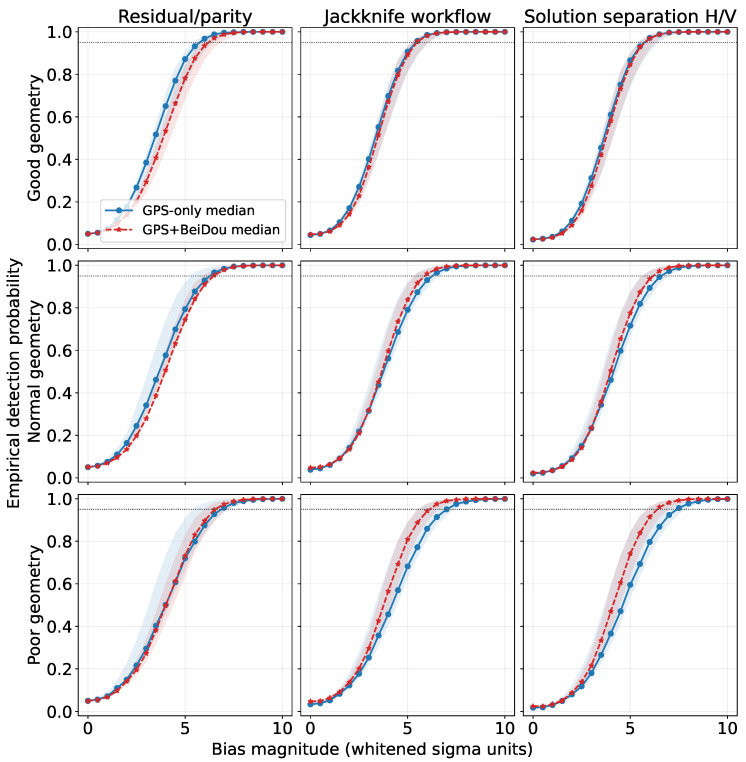
Direction-conditioned workflow detection probabilities for the six sensitivity cases. Lines show pointwise medians over common GPS fault directions, and shading shows the corresponding pointwise minimum–maximum range, not a confidence interval.

## Data Availability

The replication code and processed geometry data used to generate the validation results in this paper are openly available on GitHub at https://github.com/JasonYanxx/nav-detector-review-replication (accessed on 10 June 2026). The repository includes the detector-validation scripts, processed snapshot geometry and reproduction instructions.

## Abbreviations

GLRT	Generalized Likelihood Ratio Test
GNSS	Global Navigation Satellite System
GPS	Global Positioning System
H/V	Horizontal/Vertical
INS	Inertial Navigation System
LRT	Likelihood Ratio Test
MDB	Minimum Detectable Bias
PNT	Positioning, Navigation, and Timing
RAIM	Receiver Autonomous Integrity Monitoring
SS	Solution Separation

## Appendix A. Proof of Variance Equivalence

This appendix verifies that $\frac{\left|\ell_{q}^{⊤}A^{-1}h_{k}^{⊤}\right|}{\sqrt{S_{kk}}}$ is the same quantity as the general solution-separation standard deviation in (59). From (124),

(A1) $$\left(K-K^{\left(k\right)}\right)z=A^{-1}h_{k}^{⊤}J_{k}.$$

Using $r_{k}=S_{kk}J_{k}$ from (129) and $r_{k}=e_{k}^{⊤}r=e_{k}^{⊤}Sz$ from (14), the jackknife residual can be written as

(A2) $$J_{k}=\frac{e_{k}^{⊤}Sz}{S_{kk}},\;S_{kk}>0.$$

Substituting (A2) into (A1) gives the operator identity

(A3) $$K-K^{\left(k\right)}=\frac{A^{-1}h_{k}^{⊤}e_{k}^{⊤}S}{S_{kk}}.$$

Therefore,

(A4) $$\ell_{q}^{⊤}\left(K-K^{\left(k\right)}\right)=\frac{\ell_{q}^{⊤}A^{-1}h_{k}^{⊤}e_{k}^{⊤}S}{S_{kk}}.$$

Substituting (A4) into the general variance expression in (59) yields

(A5) $$\begin{array}{cc} \sigma_{\Delta_{k,q}}^{2} & =\ell_{q}^{⊤}\left(K-K^{\left(k\right)}\right){\left(K-K^{\left(k\right)}\right)}^{⊤}\ell_{q}\\ \; & =\frac{{\left(\ell_{q}^{⊤}A^{-1}h_{k}^{⊤}\right)}^{2}}{S_{kk}^{2}}e_{k}^{⊤}SS^{⊤}e_{k}\\ \; & =\frac{{\left(\ell_{q}^{⊤}A^{-1}h_{k}^{⊤}\right)}^{2}}{S_{kk}^{2}}e_{k}^{⊤}Se_{k}\\ \; & =\frac{{\left(\ell_{q}^{⊤}A^{-1}h_{k}^{⊤}\right)}^{2}}{S_{kk}}. \end{array}$$

The third line uses the symmetry and idempotence of the residual-maker projector S, and the last line uses $e_{k}^{⊤}Se_{k}=S_{kk}$. Taking the positive square root gives the standard deviation used before (137).

## Appendix B. Geometry Matrix

$$\left[\begin{array}{cccc} -0.174207 & 0.381103 & -0.907972 & 1\\ 0.063992 & 0.991561 & -0.112743 & 1\\ 0.355501 & -0.151365 & -0.922338 & 1\\ -0.399147 & -0.697746 & -0.594838 & 1\\ 0.556363 & 0.784017 & -0.275276 & 1\\ 0.755290 & -0.603242 & -0.256196 & 1\\ -0.983050 & 0.112353 & -0.144879 & 1\\ 0.907747 & 0.313526 & -0.278742 & 1\\ -0.441485 & -0.219277 & -0.870063 & 1\\ -0.747371 & -0.650858 & -0.133495 & 1\\ 0.545747 & -0.489533 & -0.680086 & 1 \end{array}\right]$$

## Appendix C. Geometry/DOP and Constellation-Configuration Sensitivity

This appendix extends the illustrative validation to three GPS-only geometries and their same-epoch GPS+BeiDou configurations. Figure A1 shows the corresponding GPS+BeiDou sky plots. The detailed configuration of the six cases is listed in Table A1.

**Table A1:** Geometry and matched-local MDB summaries for the sensitivity cases.

Geometry	Configuration	Epoch	*n*	*m*	*q*	PDOP	Min/Med/Max Local MDB
Good	GPS	3549	11	4	7	1.249	4.163/4.381/4.960
Good	GPS+BeiDou	3549	19	5	14	1.172	3.936/4.155/4.688
Normal	GPS	1532	8	4	4	1.831	4.434/5.240/5.528
Normal	GPS+BeiDou	1532	16	5	11	1.491	3.971/4.376/5.033
Poor	GPS	131	7	4	3	2.287	4.699/5.984/6.268
Poor	GPS+BeiDou	131	14	5	9	1.844	3.997/4.467/5.289

For each of the six configurations, every eligible single-measurement fault direction was injected separately on the common bias grid. Figure A2 reports the resulting matched specified-direction local-test MDBs. Across the good, normal, and poor GPS-only cases, the median local MDBs are 4.381, 5.240, and 5.984 whitened standard deviations, respectively, and the corresponding maxima are 4.960, 5.528, and 6.268. For the 26 common-GPS directions, adding BeiDou lowers the matched-local MDB in all 26 comparisons; the median relative changes at the three epochs are −3.51%, −11.25%, and −13.64%.

Figure A3 reports the fault-detection workflow results for the same alarm rules and configuration-specific thresholds defined in Section 7.3. Each fault direction was simulated separately. In each panel, the line is the pointwise median detection probability over the common GPS fault directions, while the shaded area is the pointwise minimum–maximum range over those directions; it is not a confidence interval. For every direction and bias magnitude, the corresponding Type II error is $1-{\hat{P}}_{D}\left(k,b\right)$. Because the workflows use different aggregation and threshold-allocation policies, the plotted curves are descriptive policy-conditioned responses and are not used to infer an intrinsic workflow ranking.
